# Isolation and quantification of polyphenolics, exploration of antioxidant, cytotoxicity, and wound healing activities of *Pithecellobium dulce* (Roxb.) Benth

**DOI:** 10.1038/s41598-025-32257-7

**Published:** 2026-01-11

**Authors:** Alaa A. Elhewehy, Ahlam M. El-Fishawy, Rasha M. Aly, Engy Mohsen, Marwa A. A. Fayed

**Affiliations:** 1 Department of Pharmacognosy, Faculty of Pharmacy, University of Sadat City, Sadat City, 32897 Egypt; 2 Pharmacognosy Department, Faculty of Pharmacy, Cairo University, Kasr El-Ainy Street, Cairo, 11562 Egypt; 3 Organic and Medicinal Chemistry Department, Faculty of Pharmacy, University of Sadat City, Sadat City, 32897 Egypt; 4 Egyptian Drug Authority, Giza, Egypt

**Keywords:** Leguminosae, *P. dulce*, HPLC-DAD, Afzelin, Fisetin 3-*O*-rhamnoside, Lignans, Natural products, Cancer

## Abstract

*Pithecellobium dulce* represent a valuable source of biologically active phytoconstituents. This study aimed to screen the methanolic extract for its different phytochemical classes, isolate the active principles from the ethyl acetate fraction, and conduct phenolic profiling using the HPLC-DAD technique. Additionally, the antioxidant, wound-healing, and cytotoxic activities were assessed in vitro. The molecular modelling (docking) studies are performed for the investigation of cervical and breast cancer cytotoxic activities for some isolated compounds. The extract phytochemical screening revealed the presence of flavonoids, alkaloids, anthraquinones, terpenes, sterols, tannins, saponins, carbohydrates, and reducing sugars. Kaempferol-3-*O*-rhamnoside (Afzelin) **(1)**, fisetin 3-*O*-rhamnoside **(2)**, and alangilignoside D **(3)** were isolated and identified from the ethyl acetate fraction using LC-MS, ^1^H and ^13^C NMR analysis. The methanolic extract showed total phenolics, flavonoids, tannins, and alkaloids of 51.44 mg GAE/g extract, 49.48 mg RE/g extract, 145.5 mg CE/g extract, and 18.62%, respectively. HPLC-DAD analysis enabled identifying and quantifying 13 phenolic acids and 7 flavonoids. The extract’s antioxidant activity was assessed using DPPH and ORAC assays, revealing an IC_50_ of 239.5 ± 8.42 µg/ml and 575.94 ± 11.30 µM TE, respectively. The extract achieved a wound closure % of 78.78 after 72 h. The cytotoxic activity was assessed against 4 cancer lines using the MTT assay, which revealed significant cytotoxic activity against the HeLa cell line, using cisplatin as a reference drug. The present investigation dealt with molecular modelling (docking) of the potent compounds showing their anticancer activities targeting cervical carcinoma HeLa cell line and breast cancer cell line MCF-7 for BCL-2 XL and EGFR. Consequently, *P. dulce* can be considered an effective natural antioxidant and cytotoxic agent.

**Supplementary Information:**

The online version contains supplementary material available at 10.1038/s41598-025-32257-7.

## Introduction

Medicinal plants have long been recognized as valuable sources of potential treatments for various human diseases. The medicinal value of these plants is attributed to their phytochemicals, including alkaloids, phenols, flavonoids, saponins, steroids, glycosides, tannins, and terpenoids. These compounds have proven effective against cancer, diabetes, inflammation, and many other disorders. The therapeutic properties of medicinal plants have spurred interest in alternative medicine, which often has fewer side effects than conventional drugs. Plant extracts have shown effectiveness in cancer therapy due to their bioactive components and many cancer treatments were derived from plants^1^.

Numerous natural bioactive compounds are known for their antioxidant activity. This ability to scavenge free radicals is crucial, as free radicals are linked to numerous life-threatening disorders. In recent decades, medicinal plants have become an important source of natural antioxidants, playing a key role in treating various human ailments. Therefore, plants with strong antioxidant activity may have the potential to treat a range of illnesses where oxidative stress is a significant factor^2^.

Cancer is the second greatest cause of mortality in both developed and developing nations^3^. In 2020, over 18.1 million cancer cases were reported globally. Lung and breast cancers are the most prevalent malignancies worldwide. Cervical cancer ranks as the fourth most prevalent cancer among women and the seventh overall in cancer incidence. As the worldwide burden of cancer continues to rise, one of the greatest public health concerns of the twenty-first century is cancer prevention^4^. Epidermal growth factor receptor (EGFR) protein expression has moderate to high levels in Cervical cancer^5^. EGFR is overexpressed in 91% of advanced cervical cancer^6^. Also overexpression of EGFR or the high activity of EGFR signal pathway has been related in breast cancer patients with increases in cell proliferation^7^. Apoptosis is known to be regulated by the BCL-2 gene regulates apoptosis, which encodes a cellular protein that inhibits apoptosis in normal cell^8^. The progression of premalignant cervical lesions to invasive malignancy is linked to the expression of BCL-2, and the prognostic value of BCL-2 as a predictor of treatment outcomes in cervical cancer have been evaluated via various studies^9^.

*Pithecellobium dulce* (Roxb.) Benth. family Leguminosae is a moderately sized perennial tree with a wide geographical distribution. Out of 100–200 species of *Pithecellobium*, *P. dulce* popularly known as “Manila tamarind”, is the only one that has expanded outside its native range. The word “dulce” indicates the pulp’s sweetness in the species’ Latin name. However, the Greek words “Pithekos” (ape) and “Lobos” (pod), which describe the real shape of the pod, are combined to form the genus^10^.

Historically, different *P.dulce* extracts have been utilized to treat various ailments in multiple nations due to several physiologically active phytoconstituents. *P. dulce* has been scientifically proven to possess numerous biological activities such as anti-inflammatory, antidiabetic, antibacterial, antiulcer, antioxidant, anticancer, hepatoprotective, antidiarrheal, cardioprotective, and nephroprotective activities^11^.

*P. dulce* fruit is consumed in various parts of India raw due to its sweet flavor and as a decoction or mixed with atole (a hot beverage made with corn flour) or agua fresca (a cold tea)^12^. The seeds can be boiled, roasted, or used as a substitute for coffee. It can also be used as a condiment to treat diabetes mellitus and stomach ulcers^13^. The infusion of leaves relieves toothaches, earaches, gallbladder, and intestinal problems. Vitamins like ascorbic acid, niacin, thiamine, and riboflavin, as well as several amino acids such as valine, phenylalanine, lysine, and tryptophan, and a few valuable minerals like Ca, Na, P, K, and Fe, are present in *P. dulce* fruits and seeds, which increase its nutritive value^14^. Given its rich nutrient profile and various medicinal uses, *P. dulce* may have potential applications in cancer prevention.

In our previous work, 67 phytoconstituents of different chemical classes mainly flavonoids and phenolic acids were identified from the methanolic extract of *P. dulce* leaves using UPLC-ESI–MS/MS technique. In addition, the extract demonstrated a neuroprotective and anti-amnesic property against scopolamine-induced cognitive decline and cerebral damage through the inhibition of acetylcholinesterase activity, reduction of dopamine and noradrenaline levels, and elevation of acetylcholine content in the brain. The extract also demonstrated antioxidant and anti-inflammatory properties^15^.

The present study was designed to explore other potentials of *P. dulce* by exploring the phytochemical constituents of the leaves’ methanolic extract through their isolation via column chromatography. Besides, phytochemical screening of various classes and profiling of phenolic compounds utilizing high-performance liquid chromatography with a diode array detector. Furthermore, the study assessed the total content of phenolics, flavonoids, tannins, and alkaloids, and assessed the biological activities for possible antioxidant, wound-healing, and cytotoxic properties. Also applying molecular modelling (docking) studies to determine the binging mode of the potential compounds with the targeted enzymes in the cytotoxic activity on both cervical cancer HeLa cell line and breast cancer cell line MCF-7.

## Materials and methods

### Chemicals, reagents, and materials

All experiments and methodologies, including plant collection, were conducted in compliance with national and international regulations as followed by the Faculty of Pharmacy, University of Sadat City, Sadat City, Egypt.

The following chemicals, solvents and reagents were used: distilled water, concentrated H_2_SO_4_, ferric chloride, Fehling’s A, B, Dragendorff’s reagent, potassium hydroxide, acetone, sodium hydroxide, methanol, ethyl ether, ethyl acetate, dichloromethane, *n*-hexane, aluminum chloride, ammonium hydroxide, dimethyl sulfoxide, cisplatin, Dulbecco’s modified Eagle’s medium, fetal bovine serum, penicillin G sodium (10.000 UI), streptomycin (10 mg), amphotericin B (25 µg) (PSA), sodium dodecyl sulfate- hydrochloric acid (SDS-HCL), Trolox, rutin, gallic acid, catechin, DPPH, APPH, acetic acid, and Na_2_CO_3_.

### Plant material

The leaves of *Pithecellobium dulce* (Roxb.) Benth. were collected in September 2020 from a private garden of Prof. Dr. Omar Ahmed Tamam in El-Rabwa City, 6th of October City, Giza, (29°54’21.6” N 30°55’27.2” E). The plant was verified by Prof. Dr. Abdel-Halim Abdel-Magley, Head of the Flora and Phytotaxonomy Research Unit at the Horticulture Research Institute in Giza, Egypt. Under reference number CAIM-218, a voucher specimen was maintained in the Flora and Phytotaxonomy Research Unit of the Horticulture Research Institute in Giza, Egypt.

### Extraction and fractionation

The leaves of *P. dulce* (2 kg) were air-dried in the shade, ground into a coarse powder, and macerated in methanol (10 L) then filtered. The maceration process was repeated multiple times until no further extraction was possible. Following full extraction, the extract was concentrated using a rotary evaporator at low temperatures (less than 40 °C) under vacuum until complete dryness. The prepared dry extract (200 gm) was liquid-liquid fractionated utilizing different organic solvents of ascending polarities (*n*-hexane, DCM, and EtOAc) to obtain the *n*-hexane fraction (52 gm), DCM fraction (11 gm), EtOAc fraction (20 gm), and the remaining aqueous fraction (50 gm). All the obtained fractions were then concentrated and kept in the refrigerator at (4 °C) for further investigation.

### Phytochemical investigation

#### Phytochemical screening

Freshly prepared *P. dulce* leaves’ extracts were tested for the presence of different classes of phytoconstituents using the different phytochemical tests^16,17^ mentioned in detail in (Table S1).

#### Isolation of compounds

The ethyl acetate fraction (20 g) previously prepared was subjected to column chromatography utilizing normal phase silica gel column mesh size (60–200 µ) and was gradually eluted using a mobile phase consisting of different ratios of *n*-hexane: dichloromethane (DCM) (1:1), and the polarity was increased by 5% until DCM 100%. Then, elution was completed using several ratios of DCM: MeOH which gradually increased by 5% until 100% MeOH. The obtained fractions were collected by TLC monitoring and sprayed with several reagents specific for different chemical classes to obtain 30 fractions. Three compounds were isolated **Compound (1)** (50 mg) was obtained as yellow crystals from the purification of fraction 8 with DCM: MeOH (9:1). **Compound (2)** (25 mg) was obtained as yellowish-brown crystals from fractions (20–30) of the Sephadex sub-column of fraction (10–12) from EtOAc silica gel column chromatography, which was subjected to the preparative TLC method using silica gel and DCM: MeOH (8.5:1.5) as a mobile phase. **Compound (3)** (36 mg) was obtained as a white amorphous powder from fractions 14–17 of the Sephadex (20 L) sub-column eluted with MeOH of fraction (10–12) from EtOAc silica gel column chromatography. The isolated compounds were detected by TLC using a UV-Vis lamp, sprayed with AlCl_3_, and *p*-anisaldehyde, and identified via LC/MS, NMR (500 MHz ^1^H NMR and 100 MHz ^13^C NMR).

#### Total phenolics (TP) and total flavonoids (TF) content

The total phenolics in the methanolic extract of *P. dulce* were analyzed using Folin–Ciocalteu and standard gallic acid according to^18^. The total phenolic content was determined by utilizing the following equation: $\:\text{Y\hspace{0.17em}=\hspace{0.17em}0.0031x\hspace{0.17em}-\hspace{0.17em}0.0564}$ (R^2^ = 0.9961) based on the calibration curve (Fig. S1A) and expressed as mg gallic acid equivalent (GAE) per g of extract (mg GAE/g extract) ± standard deviation. Additionally, the total flavonoid content was computed according to^19^ employing the aluminum chloride colorimetric technique. The results were expressed as the mg rutin equivalent (RE) per g of extract (mg RE/g extract) ± standard deviation using the following equation $\:\text{Y\hspace{0.17em}=\hspace{0.17em}0.0032x\hspace{0.17em}+\hspace{0.17em}0.0398}$ (R^2^ = 0.9981) based on the calibration curve (Fig. S1B).

#### Total condensed tannins content

The total condensed tannins were obtained by using the acidic vanillin method in a 96-well microplate^20^. A micropipette was used to transfer 25 µL of sample to a labeled well in the microplate, after which 150 µL of vanillin (4%in MeOH) and 75 µL of HCl (30%) were incorporated. After 15 min, the absorbance was determined utilizing a microplate reader at 500 nm. Different concentrations of standard catechin were prepared (125, 200.250, 300, and 400 µg/mL) in methanol. The results were expressed as mg catechin equivalent (CE) per g of the extract (mg CE/g extract) using the calibration curve (Fig. S1C) and the following equation $\:\text{Y\hspace{0.17em}=\hspace{0.17em}0.0027x-0.1231}$ (R^2^ = 0.9624)^21^.

#### Total alkaloids content

200 ml of acetic acid (10% in ethanol) was mixed with 5 g of the extract, which was then weighed into a beaker. The mixture was then covered and left for 4 h. After filtering, the extract was concentrated to one-quarter of its initial volume. Until the precipitation was complete, concentrated NH_4_OH was added dropwise to the extract. The precipitate was collected, settled, cleaned with diluted NH_4_OH, and filtered^22^. The residue was dried and weighed, and the alkaloid percentage was calculated according to the equation:

$${\%\:of\:alkaloids\:= }\text { weight of crude alkaloids } / \text { weight of the extract } \times {100}$$

#### HPLC-DAD phenolic profiling

The leaves’ powder was extracted in a conical flask using 20 mL of 2 M NaOH. N_2_ was used to flush the flask, and the stopper was installed. After 4 h of shaking the flask at room temperature, 6 M HCl was employed to correct the pH to 2. The flask was centrifuged for 10 min at 5000 rpm, then the supernatant was obtained. The phenolic compounds were extracted twice using 50 mL of (1:1) Et_2_O and EtOAc. The organic layer was taken out and evaporated at 45 °C. The resulting residue was dissolved using 2 mL MeOH. The volume of injection was 50 µL and all samples underwent filtration using the Gelman Laboratory, MI Acrodisc syringe filter (0.45 μm)^23^.

The HPLC analysis was conducted using the method described by^24^ utilizing an 1100 series liquid chromatograph from Agilent Technologies coupled with an autosampler and a diode-array detector. The employed analytical column was an Eclipse XDB-C18 (150 × 4.6 μm) and a particle size of 5 μm. Also, a (Phenomenex, Torrance, CA) C18 guard column was used. The mobile phase comprised solvents A and B (acetonitrile) and (2% acetic acid in H_2_O), respectively. The flow rate was maintained at 0.8 mL/min for the 60-minute run. The gradient program consisted of the following steps: transitioning from 100% to 85% B over 30 min, then from 85%to 50%B over 20 min, followed by a transition from 50% to 0% B in 5 min, and finally from 0%to 100%B in 5 min. The peaks were observed at 280, 320, and 360 nm. It was possible to identify the peaks by comparing their retention time and UV spectra to those of the standards.

### Biological investigation

#### Total antioxidant activity

##### DPPH assay

The DPPH (2,2-diphenyl-1-picryl-hydrazyl-hydrate) assay was performed according to previous methods^25^. In brief, a 96-well plate was used to mix 100 µL of fresh DPPH (0.1% in MeOH) and 100 µL of the extract (*n* = 3). The mixture was allowed to react in the dark at room temperature for 30 min. Then, the color intensity of DPPH was measured at 540 nm, mean ± SD are used to represent the data. The radical scavenging activity (%) was determined using the equation:

$$\it \:\text{DPPH scavenging activity} \:(\%)\:=\:[(\text{Ac}-\text{As})\:/\:Ac]\: \times \:100\:$$

where **(Ac)** represents the control absorbance and **(As)** represents the extract absorbance. A microplate reader (FluoStar Omega) was utilized to document the results. A standard solution of 100 µM Trolox in MeOH was used to prepare seven concentrations (5, 10, 15, 20, 30, 40, and 50 µM). Microsoft Excel was used to analyze the data, and Graph Pad Prism 5 was used to obtain the IC_50_ values through logarithmic conversion of the concentrations and the use of the nonlinear regression equation (Fig. S2A)^26^.

##### ORAC assay

Using the method of Liang et al.^27^ with minor modifications, an oxygen radical absorbance capacity (ORAC) assay was conducted. In brief, 12.5 µL of the prepared extract (100 µg/mL in methanol) was incubated for 30 min with 75 µL of fluorescein (10 nM) at 37 °C in a 96-well plate. Three cycles of fluorescence measurements (485 EX, 520 EM, nm) were conducted (cycle time, 90 s.). Next, 12.5 µL of recently made 2,2’-azobis (2-amidinopropane) dihydrochloride (AAPH) (240 mM) was promptly incorporated into each well. For 2.5 h, fluorescence measurements (485 EX, 520 EM, nm) were carried out (100 cycles, each 90 s) using microplate reader FluoStar Omega. The extract antioxidant activity was determined as µM Trolox equivalent using the linear regression equation $\:\text{Y= 32356.3x + 989769.9}$ (R^2^= 0.9957) (Fig. S2B). The data are presented as the means (*n* = 3) ± SD.

#### Wound healing activity

The methanolic extract of *P. dulce* leaves was investigated as a wound-healing aid^28^ as follows: Human skin fibroblast (HSF) cells obtained from Nawah Scientific Inc. (Mokatam, Cairo, Egypt) were cultured (2 × 10^5^ cells/well) onto a 12-well plate for wound scratch assays, in 5% fetal bovine serum-Dulbecco’s modified Eagle’s medium (FBS - DMEM) and then incubated in 5% CO_2_ at 37 °C. The following day, horizontal wounds were inflicted on the confluent monolayer; the plate was carefully cleaned with phosphate-buffered saline (PBS), the control wells were filled with fresh medium, and the test wells were filled with extract (50 µg/mL) in fresh media. The experiment was conducted in triplicate. An inverted microscope was used to take images periodically during the incubation period for 72 h^29^. Version 3.7 of MII Image View software was utilized to analyze wound closure and scratch width at 0, 24, 48, and 72 h. time intervals^30,31^.

The migration rate was determined using $\:\text{(Rm = }{\text{W}}_{\text{i}}\text{-}{\text{W}}_{\text{f}}\text{/t),}$ where Rm represents the cell migration rate, W_i_ represents the mean initial wound width, W_f_ represents the mean final wound width, and t represents the migration duration in hrs. The wound closure percentage was computed from $\left( {{\text{A}}{{\text{t}}_{0{\text{hr}}}} - {\text{A}}{{\text{t}}_{\Delta {\text{h}}}}/{\text{A}}{{\text{t}}_{0{\text{hr}}}}} \right) \times 100$, where At_0hr_ is the mean wound area recorded at time zero after scratching and At_Δh_ is the mean wound area determined hours after the scratch was made.

#### Cytotoxic activity

##### Cell lines

*P. dulce* leaves methanolic extract cytotoxicity was screened against four cell lines of cancer, namely, human lung cancer (A-549), human cervical carcinoma (HeLa), human osteosarcoma (Saos-2), and human breast cancer (MCF-7), and one normal cell line, human fetal lung fibroblast (WI-38) (American type culture collection, LGC Promochem, UK). The source of the cells used in this study was Global Research Labs, Nasr City, Cairo 11,528, Egypt. The IC_50_ was determined for the cell line that exhibited increased activity. The screening and IC_50_ calculations were carried out utilizing the MTT assay (3-(4,5-dimethylthiazol-2-yl)-2,5-diphenyl-2 H-tetrazolium bromide).

##### In vitro screening of cytotoxic efficacy

A-549, WI-38, Saos-2, HeLa, and MCF-7 cells were implanted in 96-well culture plates the day before the experiment was carried out. The culture plates were seeded with an average of 10,000 cells in 200 µL of Dulbecco’s modified Eagle’s medium (DMEM) mixed with FBS (10%) and PSA (1%) and incubated for 24 h at 37 °C in a 5% CO_2_ environment to achieve 70% confluence. The next day, 100 µg/L *P. dulce* extract was added and incubated with cancer cells for 48 h. Additionally, the control cells were treated with DMSO (0.1%). Cisplatin was utilized as a positive control^32^.

##### MTT assay and IC_50_ calculation

Thermo Fisher, Germany Vybrant MTT Cell Proliferation Assay Kit (cat. no.: M6494) was used to conduct the cytotoxicity assay. Using DMEM, the cells (8 × 10^3^ cells per well) were plated and incubated in 96-well culture plates for 48 h at 37 °C with 5% CO_2_. Next, 100 µL of fresh media and 20 µL of MTT solution (1 mg/mL) were added to each well. Then, the plates were incubated in the same conditions for four hours. Finally, The MTT solution was eliminated, and 100 µL of SDS-HCl was introduced to the wells. The cell viability was assessed by using a spectrophotometer (ELx 800; Bio-Tek Instruments Inc., Winooski, VT, USA) to measure the optical density at 570 nm, and cisplatin was utilized as a positive control.

Serial dilutions of the extract, including 0.01, 0.1, 1.0, 10, and 100 µg/mL, were used to treat HeLa cells. The cancer cells were subjected to the previously described cell proliferation experiment. The correlation relating to the log dose of the extract and the normal response was explained using the linear regression curve. GraphPad Prism software 9 was utilized to compute the half-maximal stimulatory concentration (IC_50_).

##### Statistical analysis

One-way ANOVA was employed for the data statistical analysis, followed by Tukey’s multiple comparison tests, the unpaired t-test to check for equality between two group averages, and the *p*-value to establish the significance level. After revision and coding, the gathered data were imported into GraphPad Prism Software 9. For parametric numerical data the mean, standard deviation (± SD), and range were employed.

### Molecular modeling and docking

#### Protein target selection and preparation

The 3-dimensional (3D) X-ray crystallographic structures of BCL-2 and EGFR proteins with PDB IDs: 2W3L and 1xkk respectively were obtained from The Research Collaboratory for Structural Bioinformatics (RCSB) protein (URL: https://www.rcsb.org*)* data library. The proposed compounds were choosing for docking study via the use of CDOCKER protocol in the Accelrys Discovery studio 2.5 program using CHARMm-based molecular dynamic (MD) scheme to dock ligands into a receptor binding site. The complexes bound to the receptor molecule, such as non-essential water molecules, including heteroatoms were removed from the target receptor molecule. Finally, hydrogen atoms were added to the target receptor molecule.

#### The docked compounds

The three LC-MS, H^1^ isolated compounds Kaempferol-3-*O*-rhamnoside (Afzelin) (1), fisetin 3-*O*-rhamnoside (2), and alangilignoside D (3) and also the top 6 compounds with the highest concentration (> 100 Concentration (µg/g)) in phenolic content in Table 3 of HPLC-DAD phenolic profiling were chosen for the docking study investigations on both PDB pockets of (2W3L, and 1XKK) for both cervical and breast cancer cell lines. The results of the docking studies were listed in Table 5.

## Results and discussion

### Phytochemical investigation

#### Phytochemical screening

*P. dulce* leaves phytochemical screening confirmed the presence of medicinally important phytochemical classes such as flavonoids, alkaloids, anthraquinones, terpenes and/or sterols, tannins, saponins, carbohydrates, and reducing sugars with the absence of cardiac glycosides.

#### Isolation of compounds

**Fig. 1:**
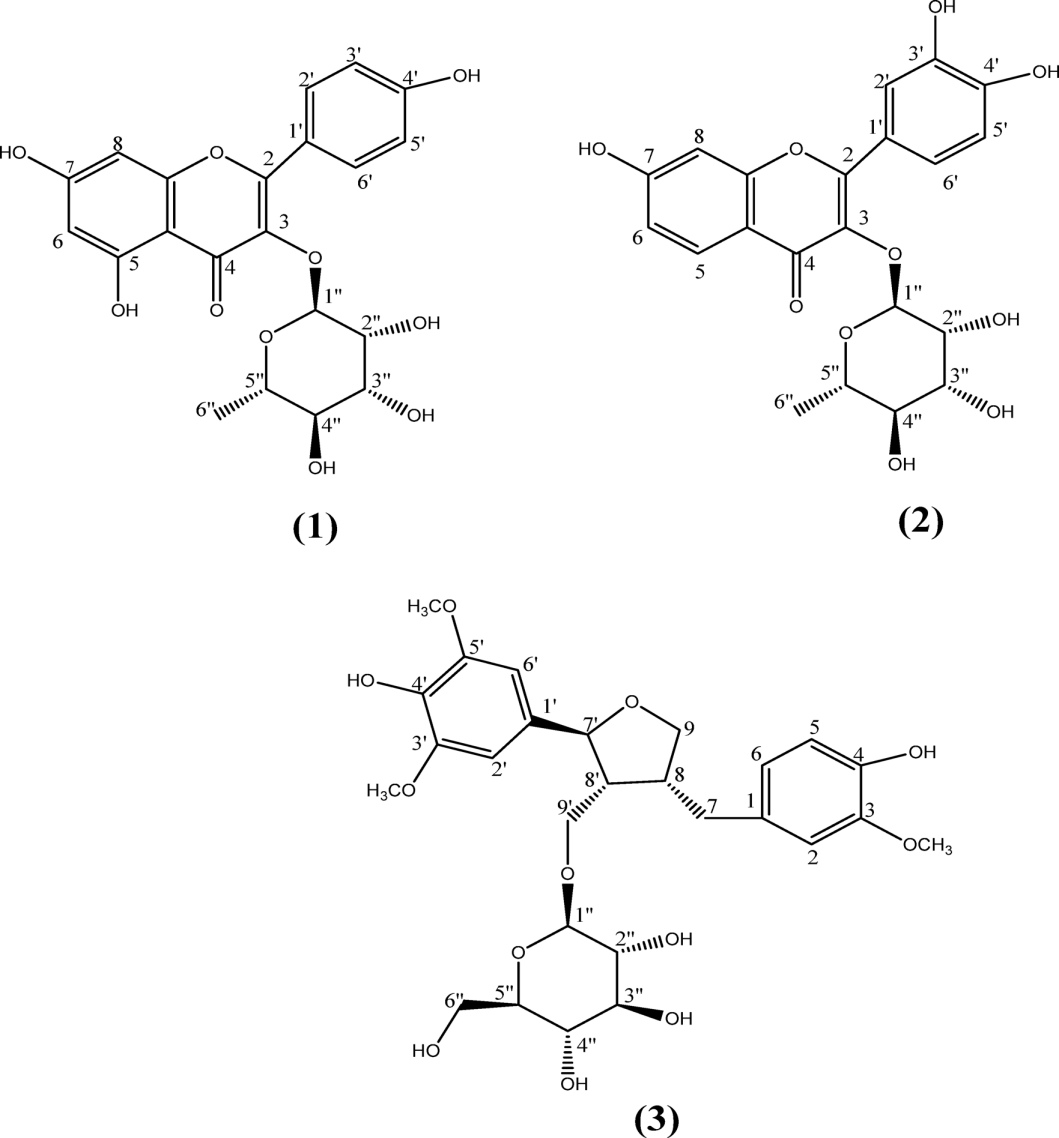
Structure of compounds isolated from *P. dulce* ethyl acetate fraction of the leaves.

Three compounds (Fig. 1) were isolated from the ethyl acetate fraction of *P. dulce* leaves: kaempferol 3-*O*-rhamnoside **(1**), fisetin 3-*O*-rhamnoside **(2)**, and alangilignoside D **(3)**. Notably, fisetin-3-*O*-rhamnoside and alangilignoside D are being reported in *P. dulce* for the first time.

**Compound (1)** (50 mg) was isolated and purified as yellow crystals. Based on the LC-MS/MS spectrum (Fig. S3) and the ^1^H (Fig. S4) and ^13^C-NMR (Fig. S5) profiles (Table 1), the molecular formula for Compound (1) was C_21_H_20_O_10_. The compound exhibited a molecular ion peak at *m/z* 433 [M + H] ^+^ & 431 [M - H]^−^ in the LC-MS spectrum^33–36^. The ^1^H-NMR spectrum displayed two aromatic hydrogen signals at δ_H_ 6.38 (br.s, 1H) and δ_H_ 6.18 (br.s, 1H), which were assigned to C-6 and C-8 hydrogens. Additionally, two signals with ‘ortho coupling’ at δ_H_ 7.71 (d, 2H, *J* = 6.5 Hz) and δ_H_ 6.89 (d, 2H, *J* = 7 Hz), these signals were attributed to H-2’,6’ and H-3’,5’, respectively. The compound was suggested to be a flavonol glycoside owing to the existence of an anomeric hydrogen signal for H-1” at δ_H_ 5.27 (br.s, 1 H). The anomeric carbon signal at δ_C_ 102.3 indicated the existence of sugar moiety, denoted as rhamnose due to the methyl group at δ_H_ 0.77 (d, 3 H, *J* = 5.8 Hz) and δ_C_ 17.9. By comparing all obtained data with those reported, Compound (1) was identified as kaempferol-3-*O*-rhamnoside (Afzelin).

**Compound (2)** (25 mg) was isolated and purified as yellowish-brown crystals. Based on the LC-MS spectrum^37^ which exhibited *m/z* 433 [M + H]^+^ molecular ion peak (Fig. S6) and the ^1^H (Fig. S7) and ^13^C-NMR (Fig. S8) profiles (Table 1)^38,39^. The compound had a molecular formula of C_21_H_20_O_10_. By comparing all obtained data with those reported, Compound (2) was identified as fisetin-3-*O*-rhamnoside which was isolated for the first time from the genus *Pithecellobium*.

**Table 1:** ^1^H (500 Hz) and ^13^C (100 Hz) -NMR data of compounds (1 & 2) isolated from the Ethyl acetate fraction of *P. dulce* leaves (DMSO-*d*_*6*_).

Compound no.	Compound (1) Afzelin		Compound (2) Fisetin 3-*O*-rhamnoside	
Carbon no.	^1^H-NMR (ppm), J in Hz	^13^C-NMR (ppm)	^1^H-NMR (ppm), J in Hz	^13^C-NMR (ppm)
2	–	157.7	–	153.6
3	–	134.7	–	139.6
4	–	178.2	–	175.8
5	12.59 (s, OH)	161.8	7.94 (d, 1 H, *J* = 2.3)	118.2
6	6.38 (brs, 1 H)	99.2	7.51 (dd, 1 H, *J* = 8.8, 2.2)	128.2
7	10.84 (s, OH)	164.7	10.49 (s, OH)	167.32
8	6.18 (brs, 1 H)	94.2	7.72 (d, 1 H, *J* = 2.1)	107.07
9	–	157.03	–	162.3
10	–	104.69	–	116.3
1ʹ	–	121	–	135
2ʹ	7.71 (d, 2 H, *J* = 6.5 Hz)	131.1	7.10 (d, 1 H, *J* = 2.1)	131.4
3ʹ	6.89 (d, 2 H, *J* = 7 Hz)	115.9	9.05 (s, OH)	147.8
4ʹ	10.18 (s, OH)	160.5	9.66 (s, OH)	150
5ʹ	6.89 (d, 2 H, *J* = 7 Hz)	115.9	7.10 (d, 1 H, *J* = 2.1)	114.5
6ʹ	7.71 (d, 2 H, *J* = 6.5 Hz)	131.1	7.99 (dd, 1 H, *J* = 7.8, 2.7)	120.05
1″	5.27 (brs, 1 H)	102.3	5.51 (d, 1 H, *J* = 2.2)	102
2″	3.96 (s)	70.9	4.16 (s, 1 H)	72.5
3″	3.08 (m)	71.14	3.79 (s, 1 H)	67.6
4″		71.69	3.63 (s, 1 H)	71.13
5″		70.62	3.17 (s, 1 H)	71.6
6″	0.77 (d, 3 H, *J* = 5.7 Hz)	17.9	1.11 (d, 3 H, *J* = 6.5 )	17.46

Compound (**3**) (36 mg) was isolated and purified as a white amorphous powder. Based on the LC-MS spectrum^40,41^ which exhibited a molecular ion peak at m/z 551 [M - H]^−^ (Fig. S9) and the ^1^H (Fig. S10), ^13^C, DEPT ^13^C NMR (Fig. S11), HMBC NMR profiles (Table 2), the compound represented a molecular formula of C_27_H_36_O_12_. The HMBC spectrum (Fig. S12) demonstrated a cross peak correlating the protons signal of the methoxy groups at δ_H_ 3.77 (s, 6H) with the carbon signal at δ_C_ 147.6 (C-3’,5’), and at δ_H_ 3.76 (s, 3H) with the carbon signal at δ_C_ 145.93 (C-3). These data confirmed that C-3’,5’, and 3 are blocked with three OCH_3_ groups. The glucose residue linkage to C 9’ was proven through the cross-peak correlation between the δ_H_ 4.36 (H-1”) signal and the δ_C_ 65.7 (C-9’) signal. By comparing all obtained data with those reported, Compound (2) was identified as alangilignoside which was isolated for the first time from this species.

**Table 2:** ^1^H (500 Hz), and ^13^C (100 Hz) -NMR data of compound (3) isolated from the Ethyl acetate fraction of *P. dulce* leaves (DMSO-*d*_*6*_).

Carbon no.	δ ^1^H-NMR (ppm), J in Hz	δ ^13^C-NMR (ppm)	DEPT 135	HMBC
1	–	137.41	C	
2	6.77 (s)	113.92	CH	
3	–	145.93	C	
4	–	144.56	C	
5	6.66 (d, 1 H, *J* = 7.8 Hz)	116.5	CH	
6	6.45 (d, 1 H, *J* = 7.7)	121.59	CH	
7a	2.52 (dd, 1 H, *J* = 11, 13)	32.61	CH_2_	
7b	2.92 (dd, 1 H, *J* = 5, 12)			
8	2.7 (m, 1 H)	44.20	CH	137.41 (C-1)
9a	3.66 (dd, 1 H, *J* = 5.88, 8)	68.26	CH_2_	
9b	3.89 (dd, 1 H, *J* = 7.9, 5.9)			
OCH_3_ (C-3)	3.76 (s, 3 H)	55.92	CH_3_	145.93 (C-3)
1ʹ	–	132.91	C	
2ʹ, 6ʹ	6.57 (s, 2 H)	104	CH	132.91 (C-1ʹ) 144.05 (C-4ʹ)
3ʹ, 5ʹ	–	147.6	C	
4ʹ	–	144.05	C	
7ʹ	4.91 (d, 1 H, *J* = 6)	86.54	CH	
8ʹ	2.48 (m, 1 H)	46.12	CH	
9ʹa	3.71 (dd, 1 H, *J* = 7.55, 10)	65.76	CH_2_	
9ʹb	4.01 (dd, 1 H, *J* = 9.47, 6.7)			
OCH_3_ (C-3ʹ,5ʹ)	3.77 (s, 6 H)	56.01	CH_3_	147.6 (C-3ʹ,5ʹ)
1″	4.36 (d, 1 H, *J* = 8)	106.79	CH	65.7 (C-9ʹ)
2″	3.21 (dd, 1 H, *J* = 7.9, 8.8)	72.61	CH	
3″	3.38 (m, 3 H)	76.63	CH	
4″		69.85	CH	
5″		73.61	CH	
6″ a	3.61 (dd, 1 H, *J* = 5, 11)	63.33	CH_2_	
6″ b	3.81 (dd, 1 H, *J* = 12, 2)			

#### Total phenolics and total flavonoids content

The total phenolics in *P. dulce* leaves methanolic extract was 51.44 ± 1.36 mg GAE/g extract. Moreover, the total flavonoids were 49.48 ± 3.6 mg RE/g extract.

#### Condensed tannins content

The total condensed tannins of the *P. dulce* leaves methanolic extract was 145.5 ± 7.6 mg CE/g extract.

#### Total alkaloids content

The bioactive total alkaloids content in *P. dulce* leaves was 18.62%.

#### HPLC-DAD phenolic-profiling

The HPLC-DAD analysis is one of the most frequently applied techniques to identify and quantify flavonoids and phenolic compounds present in plants. This approach is convenient and adaptable, offering numerous advantages such as high resolution, selectivity, sensitivity, and precision^42^.

**Fig. 2:**
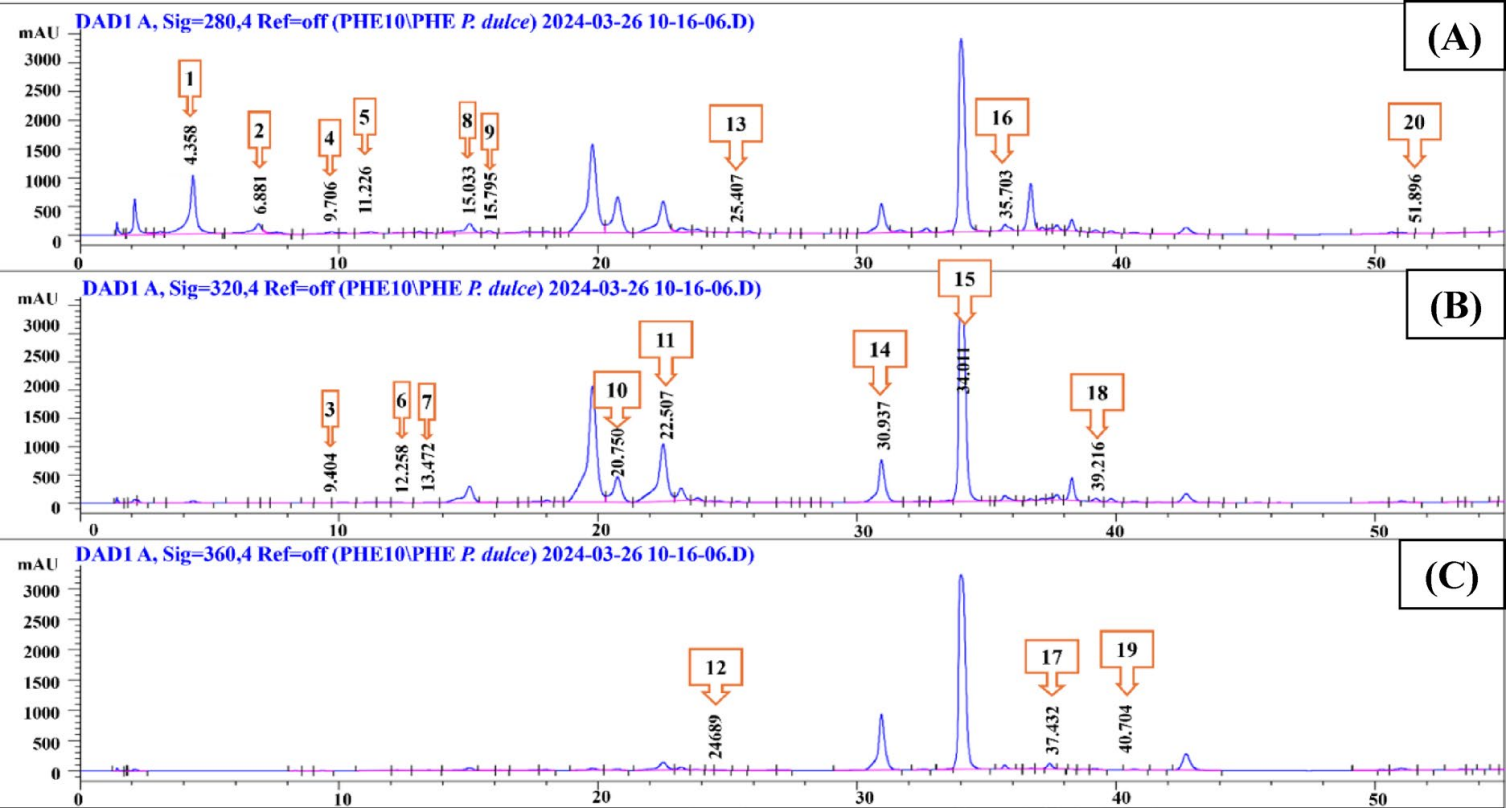
HPLC-DAD chromatogram of the methanolic extract of *P. dulce* leaves’ phenolic constituents at three different wavelengths: (**A**): λ_max_ 280, (**B**): λ_max_ 320, (**C**): λ_max_ 360.

The phenolic composition of *P. dulce* leaves methanolic extract was determined and quantified in µg/g using HPLC–DAD and twenty reference phenolic acids and flavonoids as standards. The HPLC chromatogram (Fig. 2), enabled the determination and quantification of a total of (20) phytoconstituents including (13) phenolic acids and (7) flavonoids (Table 3). Gallic acid and ferulic acid were the most abundant phenolic acids with values of 1433.70, and 941.28 µg/g, respectively. Kaempferol and apigenin showed the highest concentrations among the analyzed flavonoids with values of 370.74 and 234.46 µg/g respectively.

All the quantified compounds possess variable biological activities. Gallic and ferulic acids are among the most abundant phenolic acids reported in plants. Numerous scientific studies document their pharmacological properties, including antioxidant, anti-inflammatory, and cytotoxic activities^43,44^. Kaempferol and apigenin are prevalent flavonoid molecules found in numerous plants, exhibiting various biological functions such as anti-inflammatory, antioxidant, and cytotoxic activities through different mechanisms^45^.

**Table 3:** HPLC-DAD phenolic profiling of *P. dulce* leaves methanolic extract.

No.	*R*_t_ (min)	Identified compound	Concentration (µg/g)
1	4.3	Gallic acid	1433.70
2	6.8	Protocatechuic acid	110.36
3	9.4	Gentisic acid	24.12
4	9.7	*p-*Hydroxybenzoic acid	72.78
5	11.2	Catechin	9.42
6	12.2	Chlorogenic acid	6.48
7	13.4	Caffeic acid	270.77
8	15	Syringic acid	33.56
9	15.7	Vanillic acid	14.69
10	20.7	Ferulic acid	941.28
11	22.5	Sinapic acid	29.98
12	24.6	Rutin	20.36
13	25.4	*p-*Coumaric acid	5.70
14	30.9	Rosmarinic acid	7.37
15	34	Apigenin-7-*O*- glucoside	10.88
16	35.7	Cinnamic acid	15.10
17	37.4	Quercetin	82.05
18	39.2	Apigenin	234.46
19	40.7	Kaempferol	370.74
20	51.8	Chrysin	18.08

### Biological investigation

#### Antioxidant activity

Numerous in vitro assays exist to assess the radical scavenging activity of the methanolic plant extract. The radical scavenging effect was carried out using two different assays, the DPPH and ORAC assays due to their simplicity, reproducibility, and ability to measure different aspects of the antioxidant capacity.

The DPPH assay is commonly utilized to assess the free radical scavenging capacity of plant extracts due to its rapidity, simplicity, and cost-effectiveness^25^. On the other hand, the ORAC assay is thought to simulate the phenolics’ antioxidant activity in biological systems better than other assays as it employs biologically relevant free radicals and integrates the time and the antioxidants’ degree of activity^46^.

##### DPPH assay

The antioxidant effect of the leaves methanolic extract was evaluated utilizing the DPPH assay, shown in the initial screening stage, that the IC_50_ was between 100 and 1000 µg/mL. The dose that achieved 50% inhibition (1000 µg/mL) was serially diluted to five concentrations, revealing an IC_50_ of 239.5 ± 8.42 µg/mL (Fig. S2A). Trolox as a reference drug exhibits an IC_50_ of 24.42 ± 0.87 µM.

##### ORAC assay

The antioxidant activity of the total methanolic extract of *P. dulce* leaves was 575.94 ± 11.30 *µ*M TE/equivalent (Fig. S2B).

The results of both assays suggest that the phenolic compounds may contribute to the antioxidant activity of the *P. dulce* extract. Phenolics, which include phenolic acids, flavonoids, tannins, and the less common lignans and stilbenes, are significant secondary metabolites widely found across the plant kingdom. These metabolites play a critical role in defending against free radicals and oxidative stress. The free radical scavenging activity of the phenolic compounds is due to the presence of hydroxyl groups, where flavonoids inhibit the generation of ROS by chelating trace elements linked to free radical formation, thereby aiding in the ROS scavenging and boosting antioxidant activity^46,47^.

#### Wound healing activity

Wound healing is significantly influenced by several mechanisms such as ROS scavenging, apoptosis induction, and enzyme inhibition. These mechanisms can promote tissue repair, reduce inflammation, and enhance wound closure by targeting various cellular functions. By scavenging excessive ROS, antioxidants reduce oxidative stress, promote cell proliferation, and improve tissue repair. This helps in the formation of new blood vessels, collagen synthesis, and overall wound closure^48^.

Apoptosis helps remove inflammatory cells and damaged tissue, preventing excessive inflammation and promoting tissue remodeling. This ensures that only healthy cells contribute to tissue repair, enhancing the efficiency of wound healing^49^. Additionally, enzyme inhibition can reduce inflammation, promote collagen synthesis, and enhance tissue remodeling. This promotes wound healing by preserving the extracellular matrix’s structural integrity and facilitating cell migration and proliferation^50^.

**Fig. 3:**
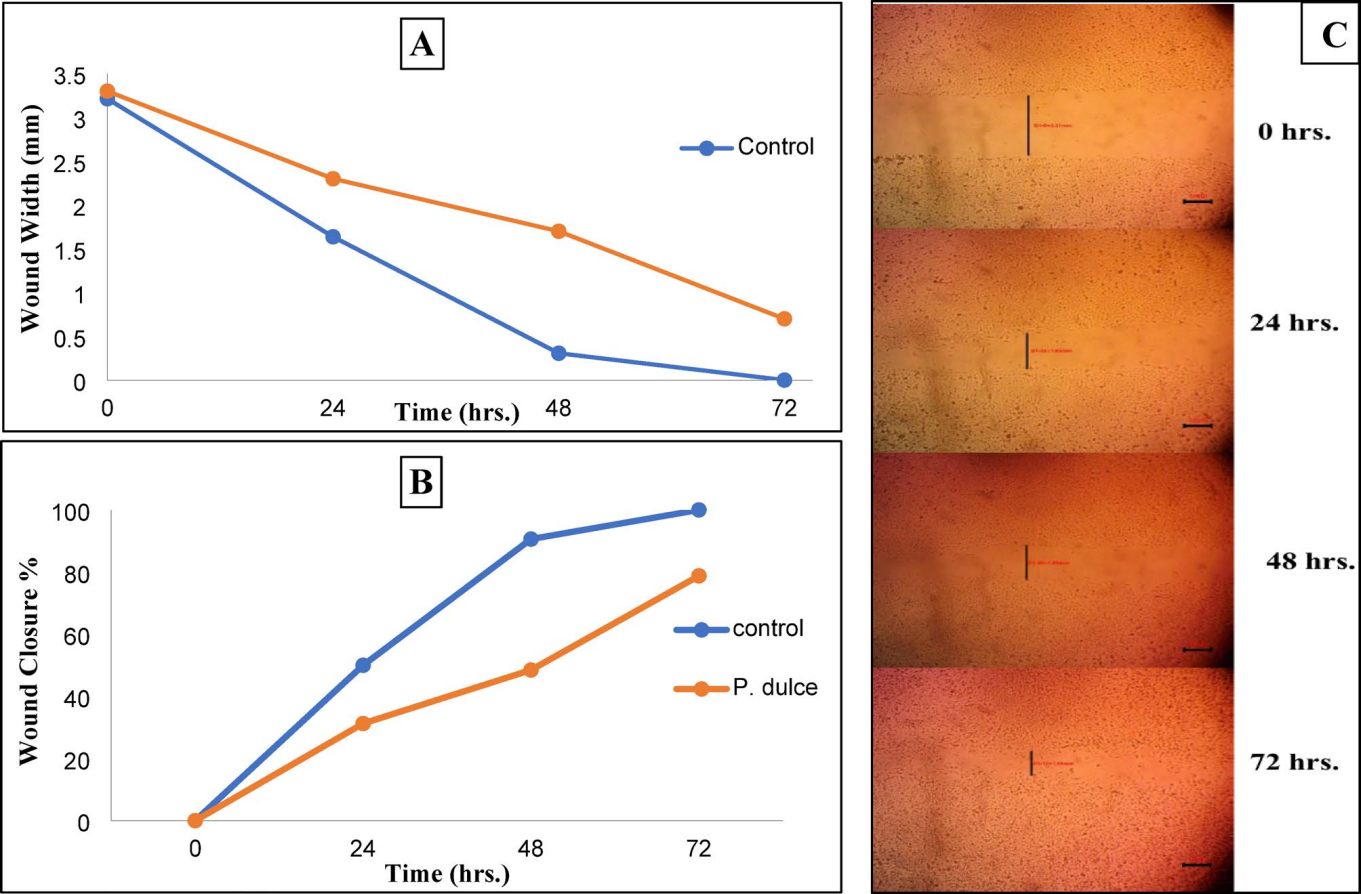
(**A**) Wound width (mm) over time (hrs.), (**B**) Wound closure %, (**C**) Microscopy images of the in vitro wound healing activity of *P. dulce* extract.

Several studies have reported the potential of many phytoconstituents such as phenolic acids, flavonoids, and alkaloids in wound treatment since they can interact in the various stages of the wound-healing pathway^51^. The wound scratch assay was employed to evaluate the wound-healing activity of *P. dulce* leaves. The average distance between the scratch edges at different time intervals (Fig. 3A and C) was used to determine the width of the wound, which diminishes as cell migration is induced, the migration rate and the wound closure percentage (Fig. 3B) were calculated (Table S2) and demonstrated in. *P. dulce* extract achieved a closure % of 78.78 after 72 h while the control (normal healing) achieved 100% closure after the same time.

#### Cytotoxic activity

Cytotoxicity in cancer therapy is significantly influenced by mechanisms such as ROS scavenging, apoptosis induction, and enzyme inhibition. Reactive oxygen species (ROS) are highly reactive molecules that cause cellular damage through oxidative stress, playing a crucial role in cancer development and progression^52^. ROS can activate various signaling pathways that control cell proliferation, survival, and apoptosis. At high concentrations, ROS can induce apoptosis in cancer cells by activating pro-apoptotic signaling pathways, such as the p53 pathway, leading to subsequent cell death. Additionally, ROS can inhibit enzymes involved in DNA repair and cell cycle regulation, causing genomic instability and uncontrolled cell growth. For instance, ROS can inhibit the activity of DNA repair enzymes, resulting in the accumulation of DNA damage^53,54^.

**Fig. 4:**
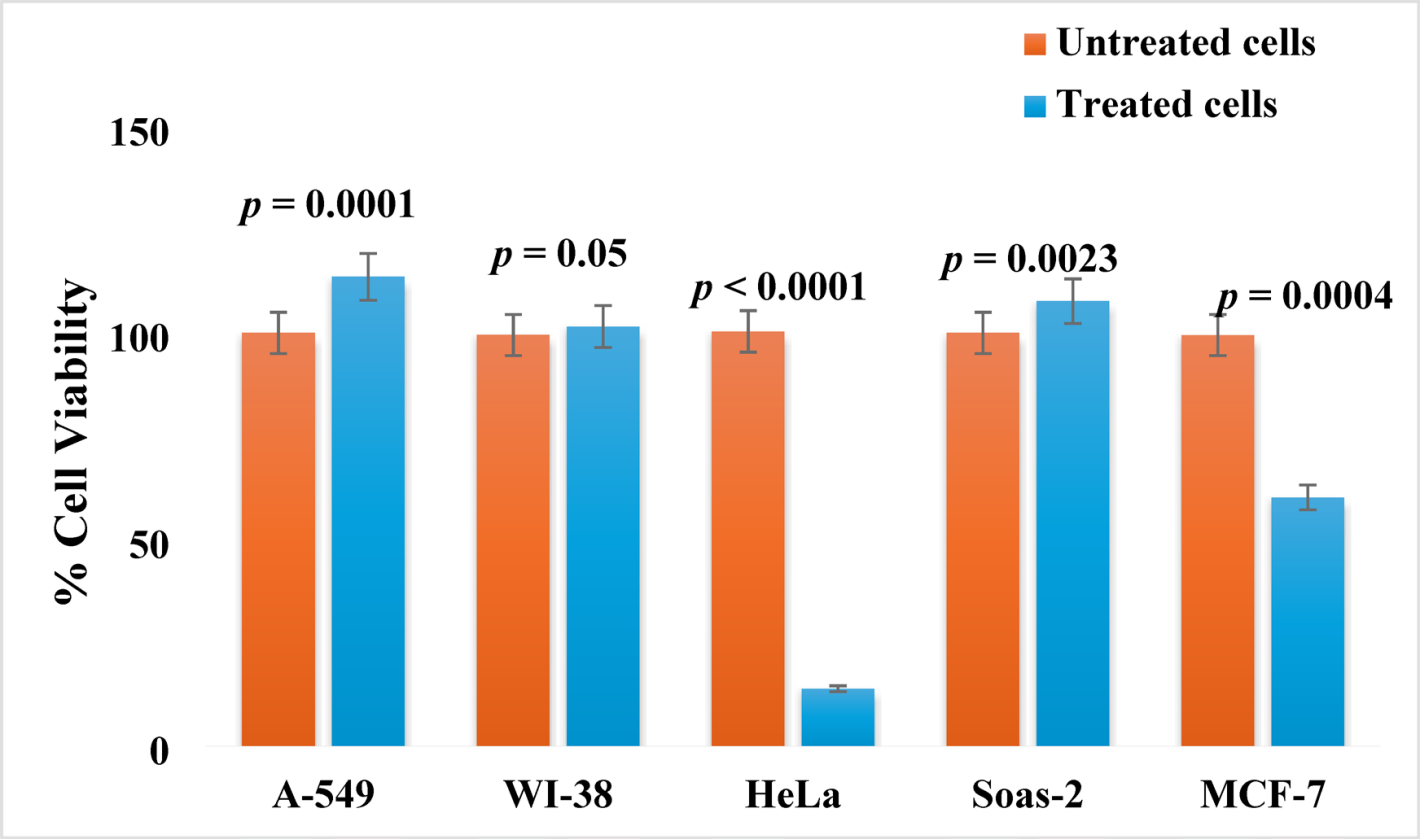
Cell viability (%) of the different cell lines untreated and treated with 100 µg/mL of *P. dulce* leaves extract for 48 h. The data are presented as the means ± SD.

The cytotoxicity of *P. dulce* leaves methanolic extract at 100 µg/mL concentration was screened against four cell lines of cancer (A-549, Saos-2, HeLa, and MCF-7), and one normal cell line WI-38 employing the MTT assay. The results revealed that the greatest effect was observed with the HeLa cell line, for which the percentage of viable cells decreased to 13.9%, followed by the MCF-7 cell line, which showed a percentage of viability of 60.28% ± 3.06. The other cell lines (A-549, WI-38, and Saos-2) showed viability percentages of 113.7% ± 1.18, 101.6% ± 0.81, and 107.8%± 0.82, respectively (Fig. 4). The unpaired independent t-test was utilized to compare the cell viability (%) between the treated and untreated cells for each cell line (Table S3). The results revealed a significant cytotoxic effect of *P. dulce* extract was detected in A-549 vs. A-549 + extract (mean difference: -13.60, *p* = 0.0001), HeLa vs. HeLa + extract (mean difference: 86.53, *p* < 0.0001), MCF-7 vs. MCF-7 + extract (mean difference: 39.27, *p* = 0.0004), and Saos-2 vs. Saos-2 + extract (mean difference: -7.70, *p* < 0.0023). Conversely, no significant difference between the treated and untreated forms was detected in the WI-38 cells (mean difference: -2.03, *p* = 0.05).

**Fig. 5:**
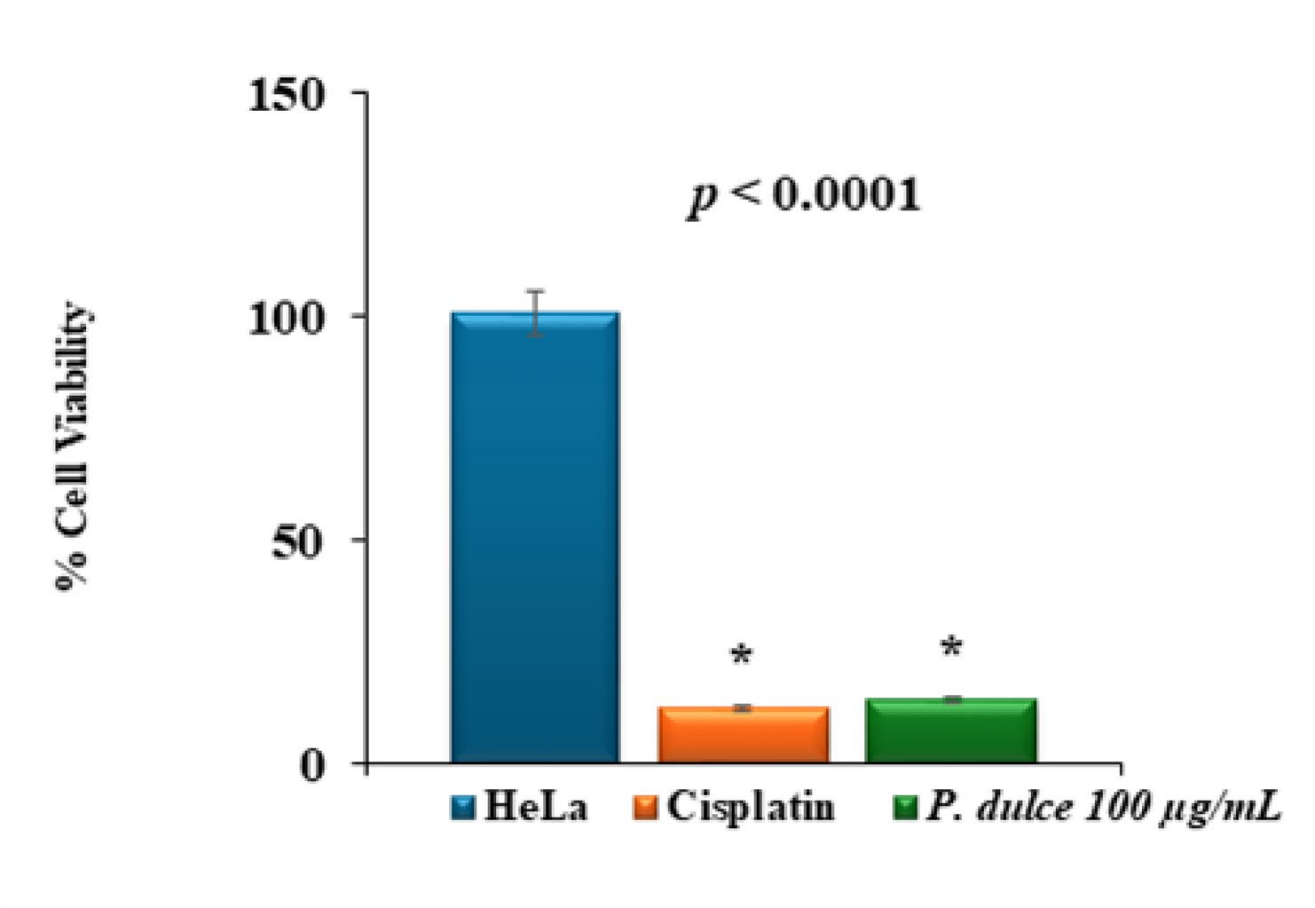
The cell viability% of the HeLa cells treated with 100 µg/mL *P. dulce* extract compared to cisplatin.

The HeLa cell line was selected as the best target for the extract effect because it showed the least viability among all the tested cancer cell lines. Cell viability was assessed after treatment with five different concentrations (0.01, 0.1, 1, 10, and 100 µg/mL) of the extract, which inhibited the HeLa cancer cells in a dose-dependent manner. For instance, at 100 µg/mL, the percentage of viable cells was 14.65% ± 3.16, whereas it was 96.17% ± 6.3 at a concentration of 0.01 µg/mL (Table 4). The IC_50_ was 2.05 µg/mL (Fig. S13). The ANOVA test (Fig. S15) was conducted to assess the significant difference between the cytotoxic effect of extract and untreated cells (negative control) as well as cells treated with standard cisplatin. The findings indicated a significant difference between the cell viability of the three tested groups (F: 929.12, *p* < 0.0001) (Fig. 5). In addition, Tukey’s multiple comparisons test (Table S3) revealed that the lowest cell viability was demonstrated in cells treated with cisplatin, and no significant difference was observed when compared to HeLa cells treated with 100 µg/mL extract (mean difference: 2.08, *p* = 0.50). As previously reported, this cytotoxic activity may result from the presence of phenolic acids, flavonoids, and alkaloids^55^.

**Table 4:** MTT assay results of the cytotoxicity of *P. dulce* leaves methanolic extract on HeLa cells.

Tested sample	HeLa (−ve control)	Cisplatin 5 µM (+ ve control)	*P. dulce* leaves methanolic extract (µg/mL)				
			0.01	0.1	1.0	10	100
Viability (%)	100.47 ± 0.50	12.55 ± 3.76	96.17 ± 6.30	81.50 ± 5.21	56.77 ± 1.29	33.27 ± 2.44	14.65 ± 3.16

Many of the compounds identified in this study have been proven to possess many biological activities. Afzelin, a flavonoid with diverse properties, presents a potential therapeutic agent owing to its anti-inflammatory, antioxidative, and anti-cancer effects, achieved by inducing apoptosis and inhibiting inflammatory pathways with fewer adverse effects than traditional therapies, and may aid in preventing treatment resistance^56^.

Furthermore, afzelin facilitates wound healing by minimizing UVB-induced cellular damage, decreasing lipid peroxidation, and preventing cell apoptosis in human keratinocytes. It also suppresses the release of pro-inflammatory mediators, thereby diminishing inflammation and facilitating tissue repair^57^.

Lignans demonstrate antioxidative properties by activating antioxidant enzymes and scavenging free radicals, thereby protecting against human LDL oxidation. They also possess anti-tumor, anti-inflammatory, anti-aging, and antimicrobial properties^58^.

Gallic acid was reported to possess cytotoxic and antitumor effects through the modification of antioxidant/pro-oxidant balance. Also, it can inhibit carcinogenesis induced by reactive oxygen species, through various mechanisms^43^. Additionally, gallic acid’s antioxidant properties may facilitate wound healing by mitigating oxidative stress at the wound site, facilitating tissue repair, and preventing infection^59^.

Ferulic acid is a potent antioxidant with the capability to scavenge free radicals and inhibit oxidative stress. It neutralizes ROS and enhances the body’s antioxidant defense systems. Furthermore, ferulic acid inhibits enzymes responsible for free radical generation and boosts the activity of scavenger enzymes. It also protects skin structures such as keratinocytes, fibroblasts, collagen, and elastin, thereby accelerating wound healing. Ferulic acid has shown cytotoxic activity against various cancer cell lines by inducing apoptosis and inhibiting cell proliferation. Additionally, ferulic acid and its derivatives have been synthesized and evaluated for their cytotoxic effects on cancer cell lines like HeLa and A-549, demonstrating promising results in inhibiting cancer cell growth and inducing cell death^60,61^.

### Molecular modelling (docking)

The polycyclic phenolic compounds containing more than 3 ring systems (Afzelin, Fisetin *O*-rhamnoside, and Alangilignoside D) show the highest -CDOCKER Interaction energy near to or higher than the main enzyme ligand in both 2W3L and 1XKK PDB pockets which higher than the tricyclic phenolic compounds (Kaempferol and Apigenin), while the monocyclic phenolic compounds show the lowest scores (ferulic acid, caffeic acid, Gallic acid and Protocatechuic acid) but still have good fitting and binding within the pocket of the targeted proteins. This investigates that the more polycyclic phenolic compounds that mimic the main reference ligands’ structures, the better binding mode, docking, H-bonds and hydrophobic interaction with the enzymes’ binding pocket. The results of the docking studies were listed in the following Table 5.

**Table 5:** Molecular Docking scores (C-Docker interaction energy) for 2W3L and 1XKK enzymes.

Compound name	2W3L C-Docker interaction energy	1XKK C-Docker interaction energy
Ligand of 2W3L: Phenyl Tetrahydroisoquinoline amide	43.8862	
Ligand of 1xkk: Lapatinib		69.8465
Afzelin	38.4124	58.7544
Fisetin *O*-rhamnoside	37.8179	59.1423
Alangilignoside D	44.4558	57.2688
Gallic acid	20.7058	24.627
Ferulic acid	25.4504	28.4905
Kaempferol	30.0543	40.4677
Caffeic acid	24.355	25.6133
Apigenin	28.0144	38.4715
Protocatechuic acid	18.7929	22.6784

#### Docking with PDB pocket (2W3L): Fig. 6-AI–AVIII)

**Fig. 6:**
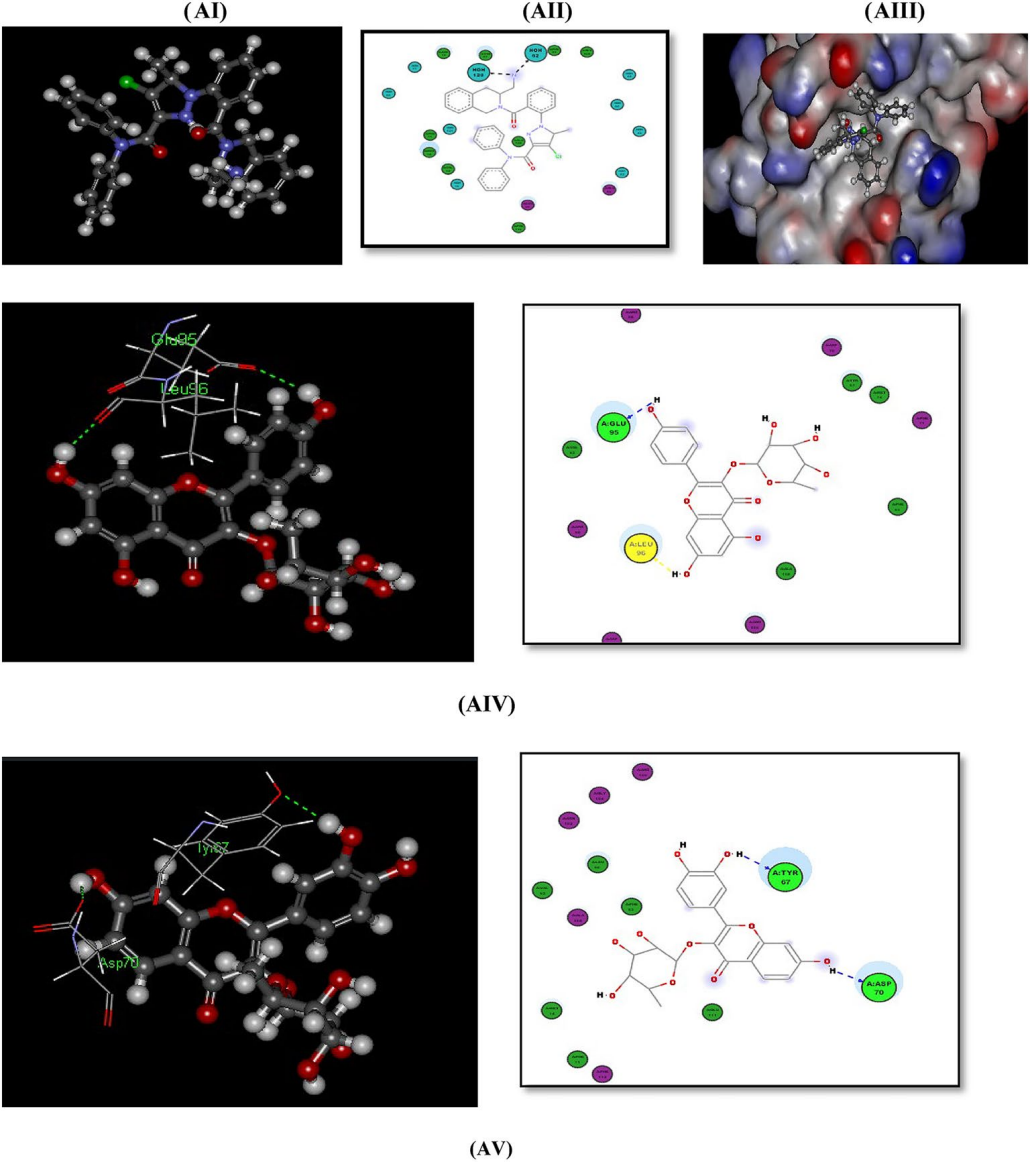

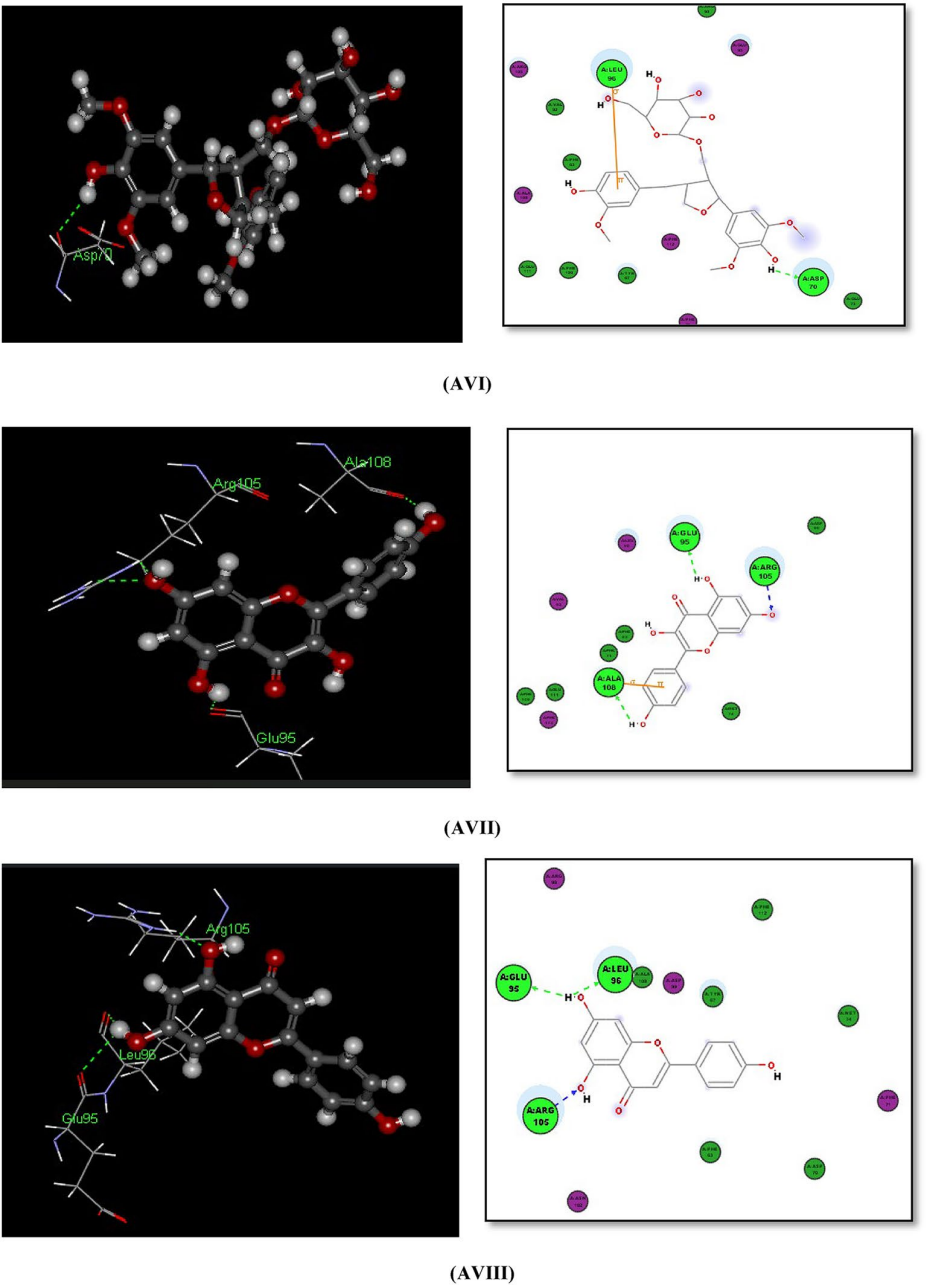
(**AI**), (**AII**), and (**AIII**): 3D and 2D Docking and the surface picture of the co crystalline Ligand Phenyl Tetrahydroisoquinoline Amide in BCL-2 XL(PDB: 2W3L). (**AIV**): Docking of afzelin within 2W3L (3D and 2D): 1 H-Bond with GLU95 and 1 H-Bond with LEU96 amino acids. (**AV**): Docking of Fisetin *O*-rhamnoside within 2W3L (3D and 2D): 1 H-Bond with TYR67 and 1 H-Bond with ASP70 amino acid. (**AVI**): Docking of Alangilignoside D within 2W3L (3D and 2D): 1 H-Bond with ASP70 amino acid. (**AVII**): Docking of kaempferol within 2W3L (3D and 2D): 1 H-Bond with GLU95, 2 H-Bonds with ARG105 and 1 H-Bond with ALA108 amino acids. (**AVIII**): Docking of apigenin D within 2W3L (3D and 2D): 1 H-Bond with GLU95, 1 H-Bond with LEU96 and 1 H-Bond with ARG105 amino acids.

The BCL-2 XL and Phenyl Tetrahydroisoquinoline amide Complex (PDB id: 2W3L) shows the fitting of the reference ligand (Phenyl Tetrahydroisoquinoline amide) in the BCL-2 XL pocket with -CDOCKER interaction energy of (43.4124). This reference ligand contains 6 ring systems that have good fitting within the pocket of the enzyme, (Fig. 6-AI-6-AIII)).

The polycyclic phenolic compound Alangilignoside D that has 4 ring systems (more than 3 ring systems that mimic the reference ligand structure) shows -CDOCKER interaction energy of (44.4558) which is greater than that of the main reference ligand dicked in 2W3L. Also, Afzelin and Fisetin *O*-rhamnoside show the -CDOCER Interaction energy of (38.4124 and 37.8179) respectively which are near to that of main ligand in 2W3L. These polycyclic compounds with 4 ring systems perform H-bonds with in the binding site and supposed to perform more hydrophobic interactions and better fitting within it due to its polycyclic structure which show structure similarity to the main reference ligand, (Fig. 6-AIV-AVI)).

The tricyclic phenolic compounds (Kaempferol and Apigenin) show the -CDOCKER Interaction energy of (30.0543 and 28.0144) respectively which are > 63% that of main ligand in 2W3L and lower than that of polycyclic compounds having 4 ring systems, but still form H-bond interactions within the BCL-2 XL pocket (2W3L) with good fitting manner in the binging pocket, (Fig. 6aVII-6aVIII).

These scores are followed by that of the monocyclic phenolic compounds (Ferulic acid, Caffeic acid, Gallic acid and Protocatechuic acid) that show the lowest -CDOCKER interaction energy scores (25.4504, 24.355, 20.7058 and 18.7929, respectively) but these scores are considered in a good range of (58% to 42%) that of the -C-DOCKER interaction energy of main ligand docked in 2W3L, and these compounds still perform 3 H-bond interactions within the BCL-2 XL pocket (2W3L) with good fitting to the enzyme pocket, (Figures S16-S19).

#### Docking with PDB pocket (IXKK): Fig. 7AI–7AVIII

**Fig. 7:**
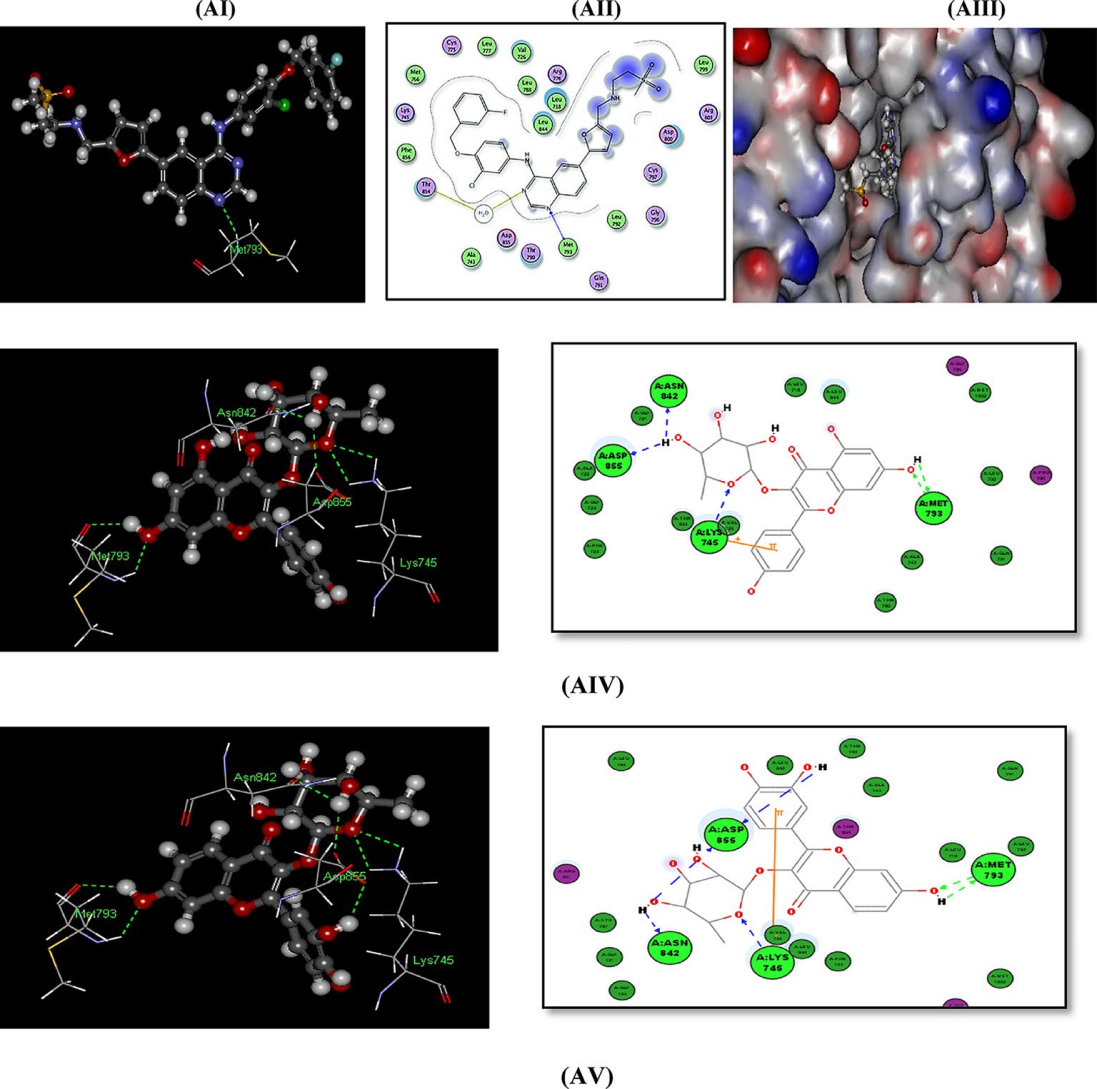

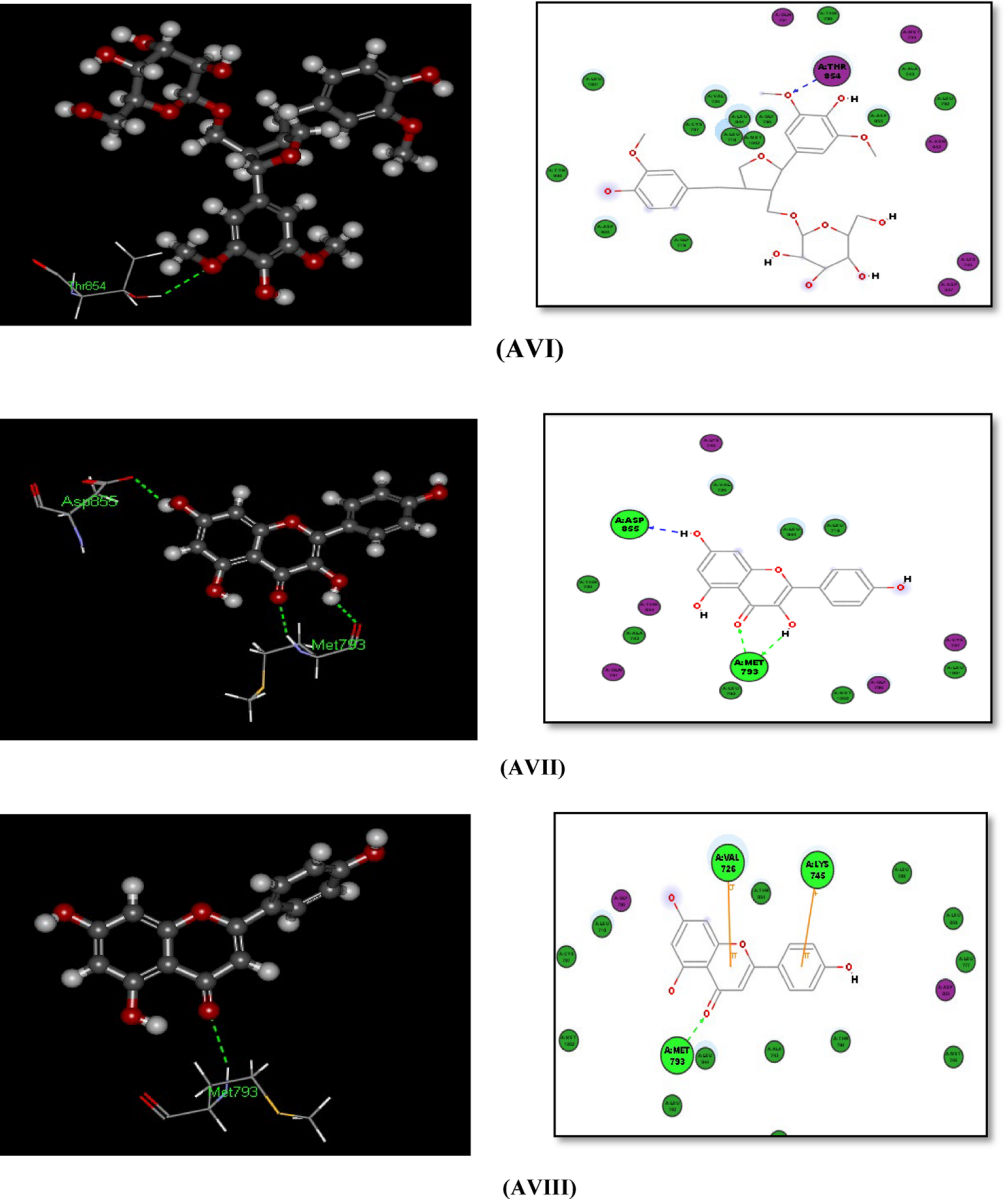
(**AI**), (**AII**) and (**AIII**): Docking of the co crystalline ligand lapatinib and 2D Docking of Lapatinib within the ATP binding site of EGFR^62^. (**AIV**): Docking of afzelin within 1XKK (3D and 2D): 2 H-Bonds with MET793, 2 H-Bonds with Lys745, 1 H-Bond with ASP855, and 1 H-Bond with ASN842 amino acids. (7-AV): Docking of Fisetin *O*-rhamnoside within 1XKK (3D and 2D): 2 H-Bonds with MET793, 2 H-Bonds with Lys745, 2 H-Bonds with ASP855, and 1 H-Bond with ASN842 amino acids. (**AVI**): Docking of Alangilignoside D within 1XKK (3D and 2D): 1 H-Bond directly with THR854 amino acid (not via H_2_O Molecule). (**AVII**): Docking of Kaempferol within 1XKK (3D and 2D): 2 H-Bonds with MET793 and 1 H-Bond with ASP855 amino acids. (**AVIII**): Docking of Apigenin D within 1XKK (3D and 2D): 1 H-Bond with MET793 amino acid.

The EGFR and Lapatinib Complex (PDB id: 1XKK) shows the fitting of the reference ligand Lapatinib in the EGFR pocket with -CDOCKER interaction energy of (69.8465). The reference ligand Lapatinib contains 5 ring systems that have good fitting within the pocket of the enzyme and performs 1 H-BOND with EGFR binding site amino acid MET 793 and also within THR 854 via H_2_O molecule in the binding pocket, (Fig. 7-AI-AIII).

The polycyclic phenolic compounds containing 4 ring systems Fisetin *O*-rhamnoside, Afzelin, and Alangilignoside D show -CDOCKER Interaction energy of (59.1423, 58.7544, and 57.2688) respectively which are near to that of main reference compound lapatinib in EGFR binding site 1XKK. These compounds perform H-bonds with in the binding site: Fisetin *O*-rhamnoside, Afzelin perform 7-H-bonds and 6-Hbonds, respectively within the binding site including 2 H-bonds with in MET793 amino acid that mimic the binding mode of the main reference ligand Lapatinib which perform 1 H-bond with MET793, while Alangilignoside D perform H-bond within THR 854 amino acid but directly via the OCH_3_ moity (not via H_2_O molecule) that mimic the binding mode of the main reference ligand Lapatinib that performs H-bond within THR854 but via H_2_O molecule, (Fig. 7-AIV-VI).

The tricyclic phenolic compounds (Kaempferol and Apigenin) show the -CDOCKER Interaction energy of (40.4677 and 38.4715) respectively which are > 55% that of main ligand in 1XKK and lower than that of the polycyclic phenolic compounds having more 4 ring systems, but still form H-bond interactions within the EGFR pocket (1XKK) including at least 1 H-bond with in MET793 amino acid that mimic the binding mode of the main reference ligand Lapatinib, with good fitting manner in the binging pocket, (Fig. 7-AVII-AVIII)).

Although the monocyclic phenolic compounds (Ferulic acid, Caffeic acid, Gallic acid and Protocatechuic acid) show the lowest -CDOCKER interaction energy scores within the EGFR pocket (1XKK) (28.4905, 25.6133, 24.627 and 22.6784, respectively) but these scores are considered in a range of (41% to 32%) of the -C-DOCKER interaction energy of that of main ligand Lapatinib in 1XKK, and these compounds still perform H-bond interactions within MET 793 of the EGFR pocket (1XKK) that mimic the binding mode of the main reference ligand Lapatinib, with good fitting to the enzyme pocket, (Figures S.20-S.23).

## Conclusion

The phytochemical screening of the *P. dulce* (Roxb) Benth. leaves methanolic extract revealed the presence of flavonoids, alkaloids, anthraquinones, terpenes, sterols, tannins, saponins, carbohydrates, and reducing sugars. Thirteen phenolic acids and seven flavonoids were recognized and quantified using HPLC-DAD analysis. In addition, three compounds were isolated from the ethyl acetate fraction, and it is noteworthy that the identified compounds fisetin 3-*O*-rhamnoside **(2)**, and alangilignoside D **(3)** are the first to be identified in *P. dulce*. Besides, the leaves’ methanolic extract exhibited in vitro antioxidant activity and antitumor effect against different cell lines, mainly the cervical carcinoma (HeLa) in a dose-dependent manner. The extract achieved a wound closure percentage of 78.78 after 72 h. The presence of phenolic acids, flavonoids, and tannins is suggested to be responsible for these activities. According to the molecular docking that discovers the binding mechanism and interaction between the proposed potential compounds with in the BCL-2 XL (PDB: 2W3L) and EGFR (PDB: 1XKK) that are overexpressed in cervical carcinoma Hela cell line and the breast cell line MCF-7, the more polycyclic phenolic compounds that mimic the main reference ligands structures, the better binding mode, docking, and H-bonds and hydrophobic interaction with the enzymes’ binding pocket as 4 ring systems polycyclic phenolic compounds (Afzelin, Fisetin *O*-rhamnoside, and Alangilignoside), followed by tricyclic phenolic compounds (Kaempferol and Apigenin) and then the monocyclic phenolic compounds (Ferulic acid, Caffeic acid, Gallic acid and Protocatechuic acid). Future research, in vivo validation, nanoparticle formulations, or clinical translation is essential to explore the full potential of each phytoconstituent present in the extract and for enhanced therapeutic potential.

## Supplementary Information

Below is the link to the electronic supplementary material.

Supplementary Material 1

## Data Availability

This article and its supplementary information file include all data generated and analyzed during this study.

## Abbreviations

A-549: Human lung cancer
APPH: 2,2′-azobis (2-amidinopropan) dihydrochloride
CE: Catechin equivalent
DMEM: Dulbecco’s Modified Eagle Medium
DPPH: 2,2-Diphenyl-1-picryl-hydrazyl-hydrate
FBS: Fetal bovine serum
GAE: Gallic acid equivalent
GSH: Glutathione
HeLa: Human cervical carcinoma
HPLC-DAD: High-performance liquid chromatography with diode-array detector
HSF: Human skin fibroblast
MCF-7: Human breast cancer
ORAC: Oxygen radical absorbance capacity
PSA: Penicillin G sodium, streptomycin, amphotericin B
RE: Rutin equivalent
ROS: Reactive oxygen species
Saos-2: Human osteosarcoma
SDS-HCL: Sodium dodecyl sulfate with hydrochloric acid
TE: Trolox equivalent
WI-38: Human fetal lung fibroblast
