# Supplementary material for: Isolation and quantification of polyphenolics, exploration of antioxidant, cytotoxicity, and wound healing activities of Pithecellobium dulce (Roxb.) Benth
Source: Sci Rep. 2026 Jan 11;16:1338. doi: 10.1038/s41598-025-32257-7 (PMC12795817; doi:10.1038/s41598-025-32257-7)
Supplement: Supplementary file 1 — Supplementary Material 1 [file 41598_2025_32257_MOESM1_ESM.pdf]

Isolation and quantification of polyphenolics, exploration of antioxidant, cytotoxicity, and wound healing activities of *Pithecellobium dulce* (Roxb.) Benth.

Alaa A. Elhewehy[1], Ahlam M. El-fishawy[2], Rasha M. Aly[3, 4], Engy Mohsen[2†*], Marwa A.A. Fayed[1†*]

[1]Department of Pharmacognosy, Faculty of Pharmacy, University of Sadat City, Sadat City 32897, Egypt.

[2] Pharmacognosy Department, Faculty of Pharmacy, Cairo University, Kasr El-Ainy Street, Cairo 11562, Egypt.

[3] Organic and Medicinal Chemistry Department, Faculty of Pharmacy, University of Sadat City, Sadat City 32897, Egypt.

[4] Egyptian Drug Authority, Giza, Egypt.

https://orcid.org/0000-0001-9622-2932, https://orcid.org/0000-0003-4925-7395,

https://orcid.org/0009-0007-5723-9476, https://orcid.org/0000-0001-5609-7436,

†Both authors contributed equally.

[*]**Corresponding authors**:

Engy Mohsen, Pharmacognosy Department, Faculty of Pharmacy, Cairo University, Kasr El-Ainy Street, Cairo 11562, Egypt.

Email: engy.mohsen@pharma.cu.edu.eg

Marwa A.A. Fayed, Department of Pharmacognosy, Faculty of Pharmacy, University of Sadat City, Sadat City 32897, Egypt.

Email: marwa.fayed@fop.usc.edu.eg

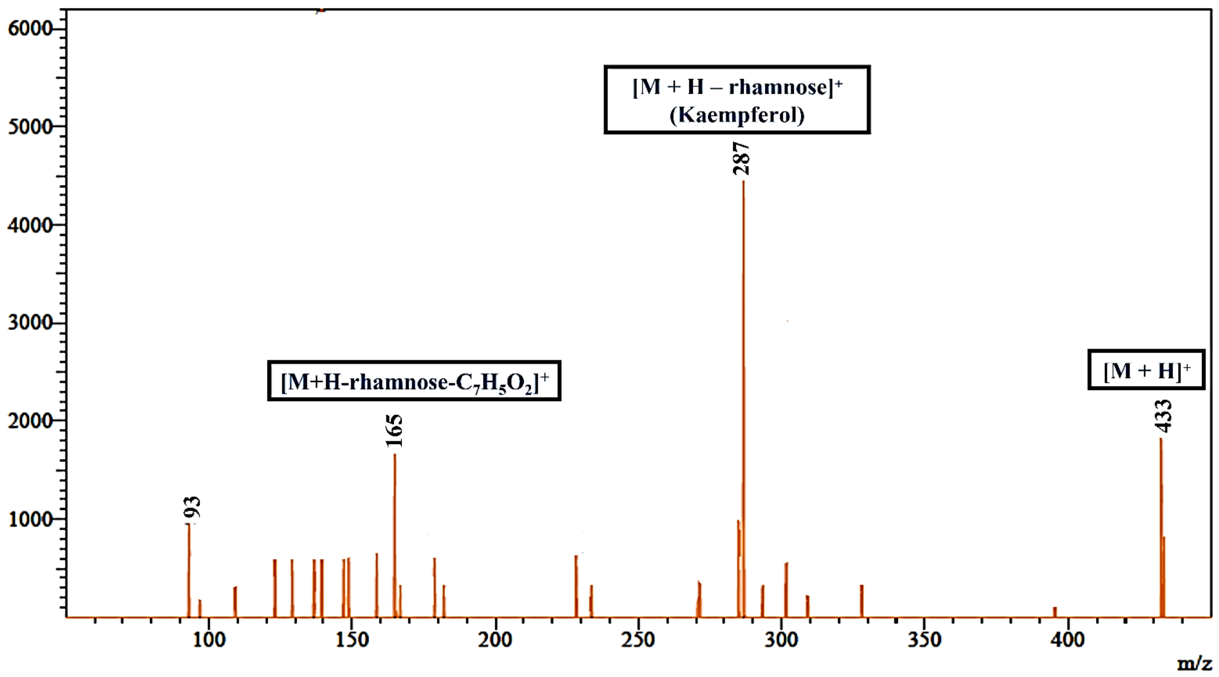

➢ **Fig. S.1:** UPLC-ESI-MS/MS chromatogram of Compound **1** (**Kaempferol-3-*O*-rhamnoside).**

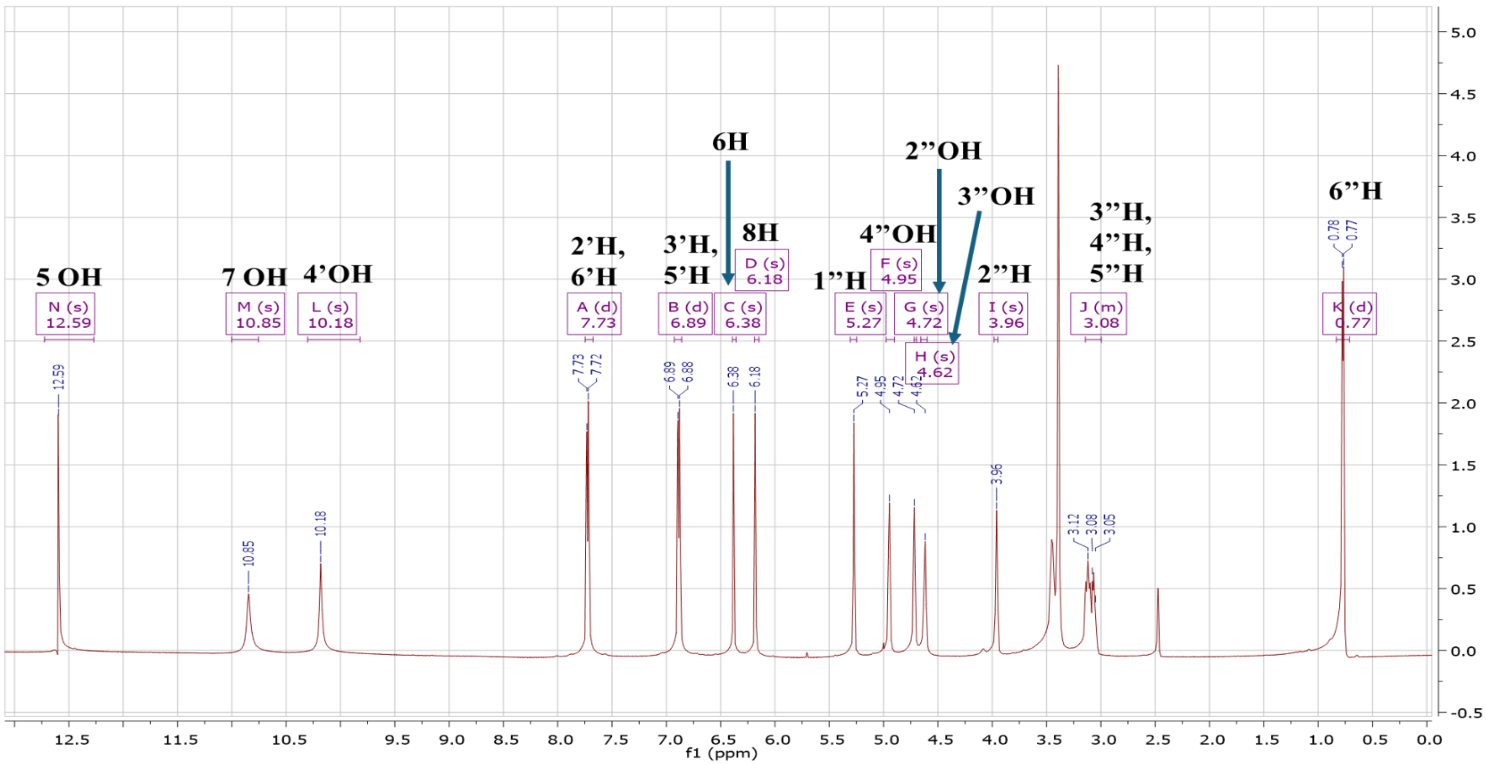

➢ **Fig. S.2:** ^{1}H NMR spectrum of Compound **1** (**Kaempferol-3-*O*-rhamnoside).**

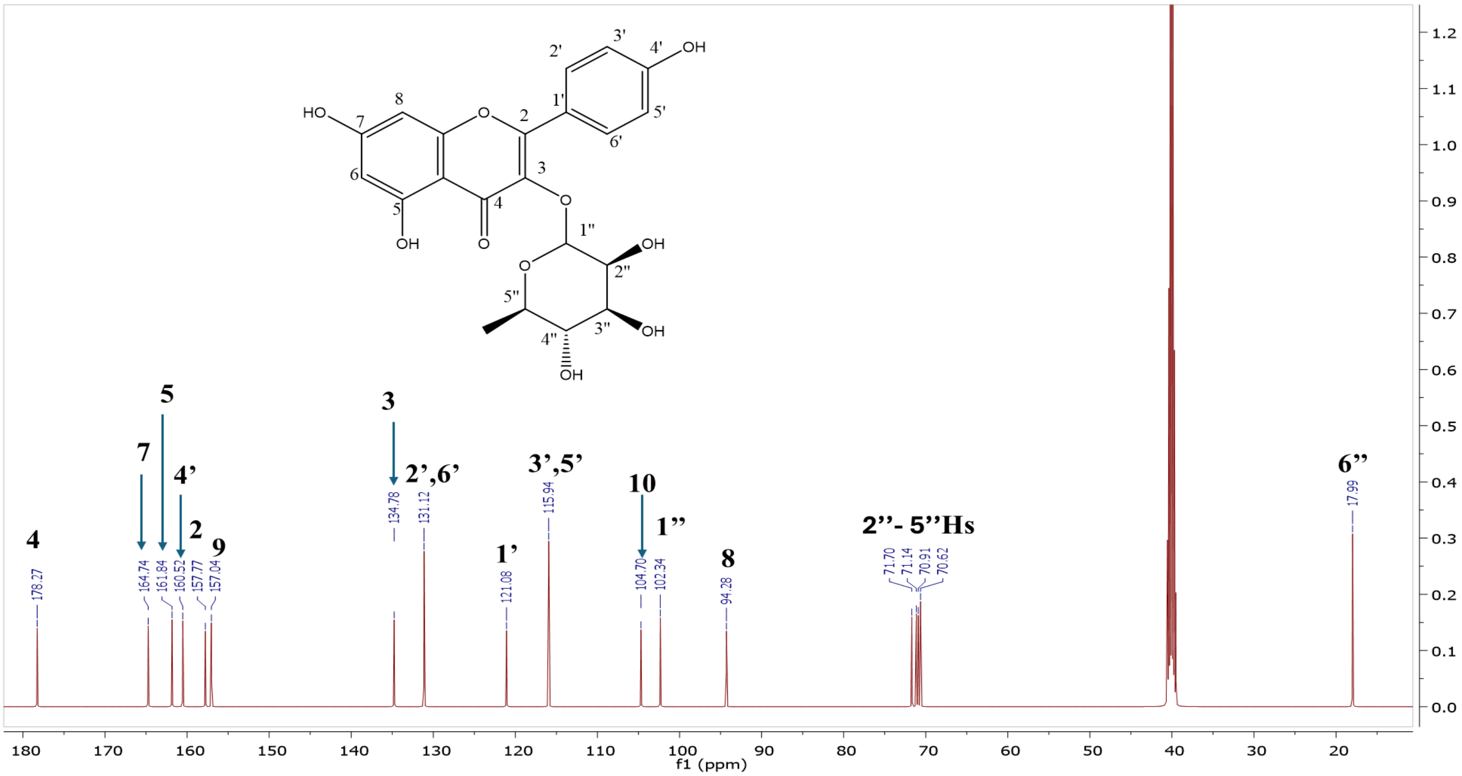

➢ **Fig. S.3:** ^{13}C NMR spectrum of Compound **1** (**Kaempferol-3-*O*-rhamnoside).**

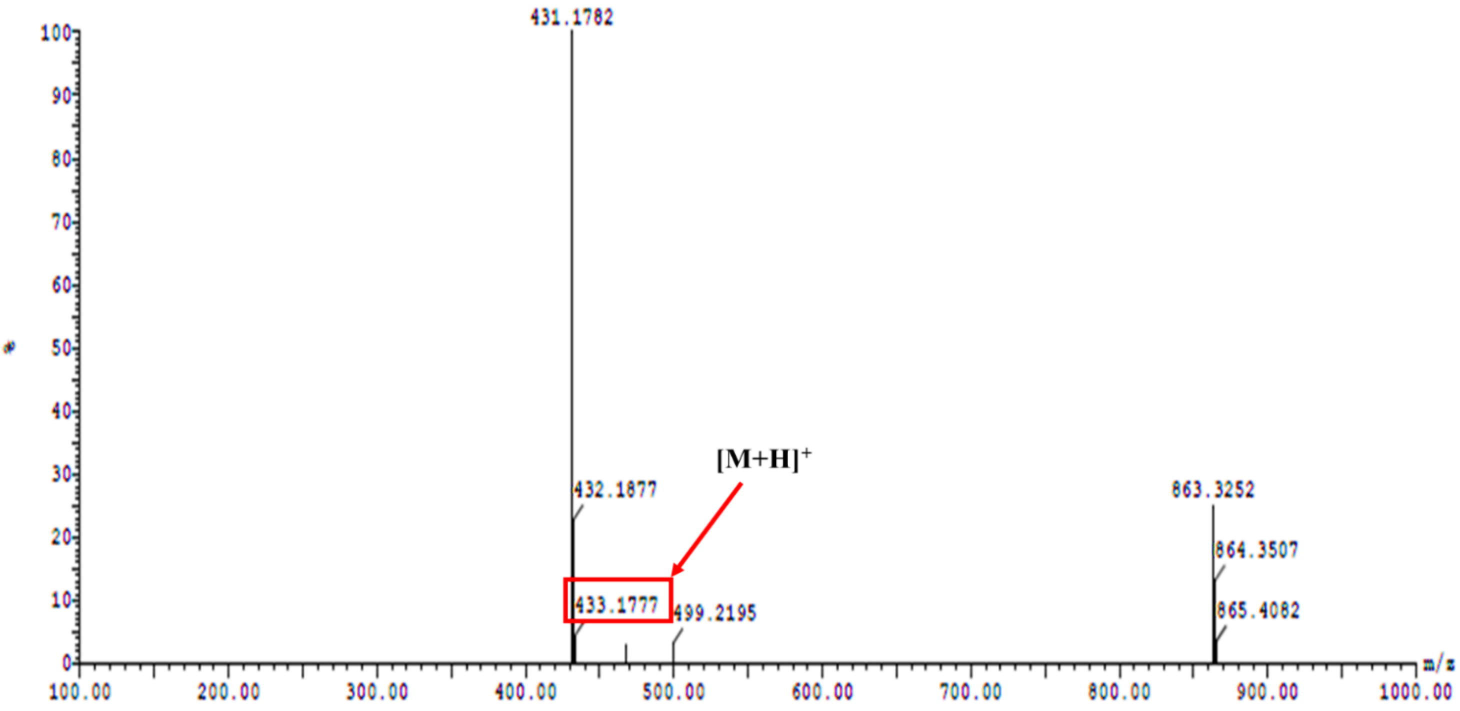

➢ **Fig. S.4:** UPLC-ESI-MS chromatogram of Compound **2** (**Fisetin-3-*O*-rhamnoside).**

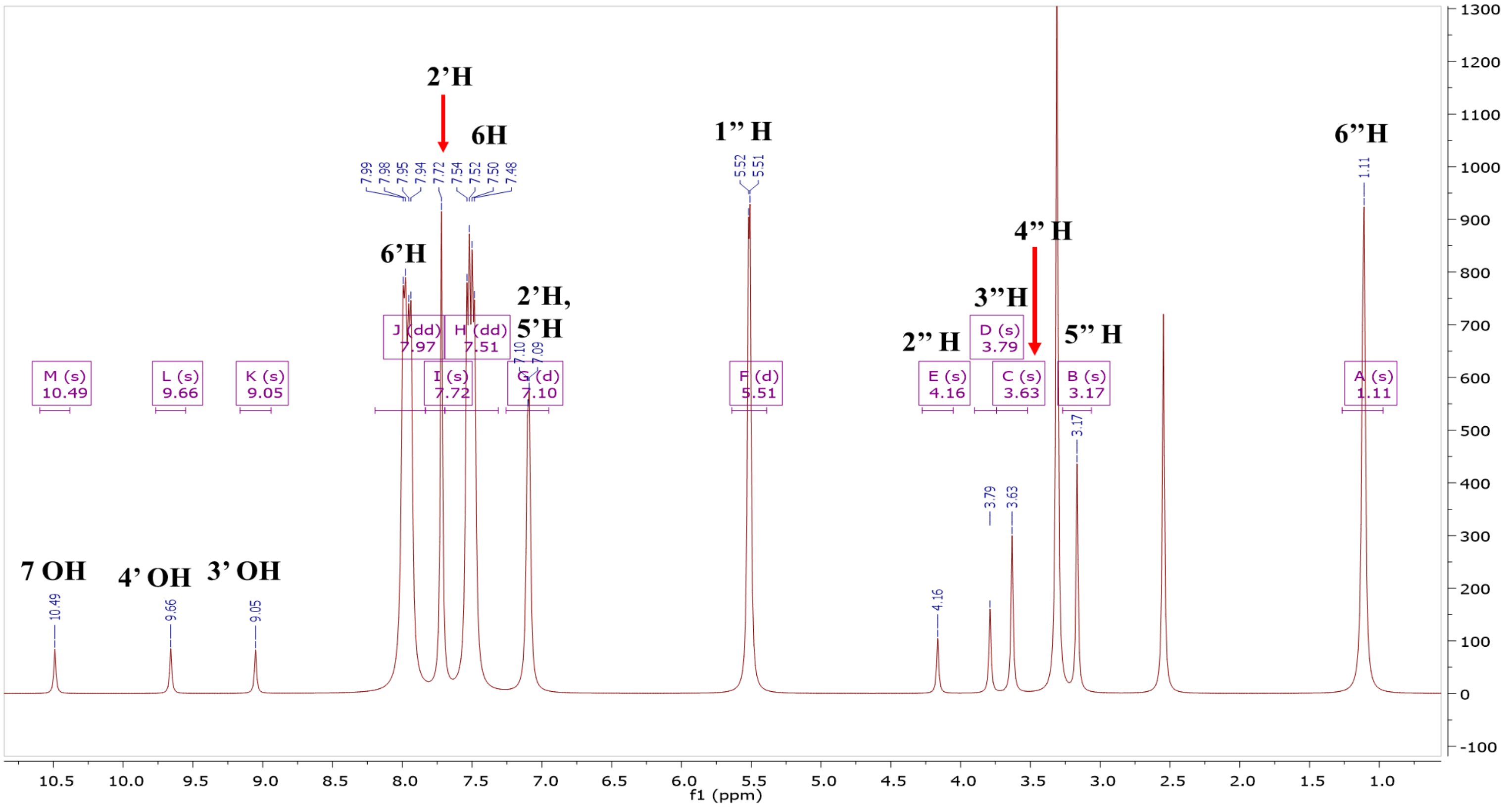

➢ **Fig. S.5:** ^{1}H NMR spectrum of Compound **2** (**Fisetin-3-*O*-rhamnoside).**

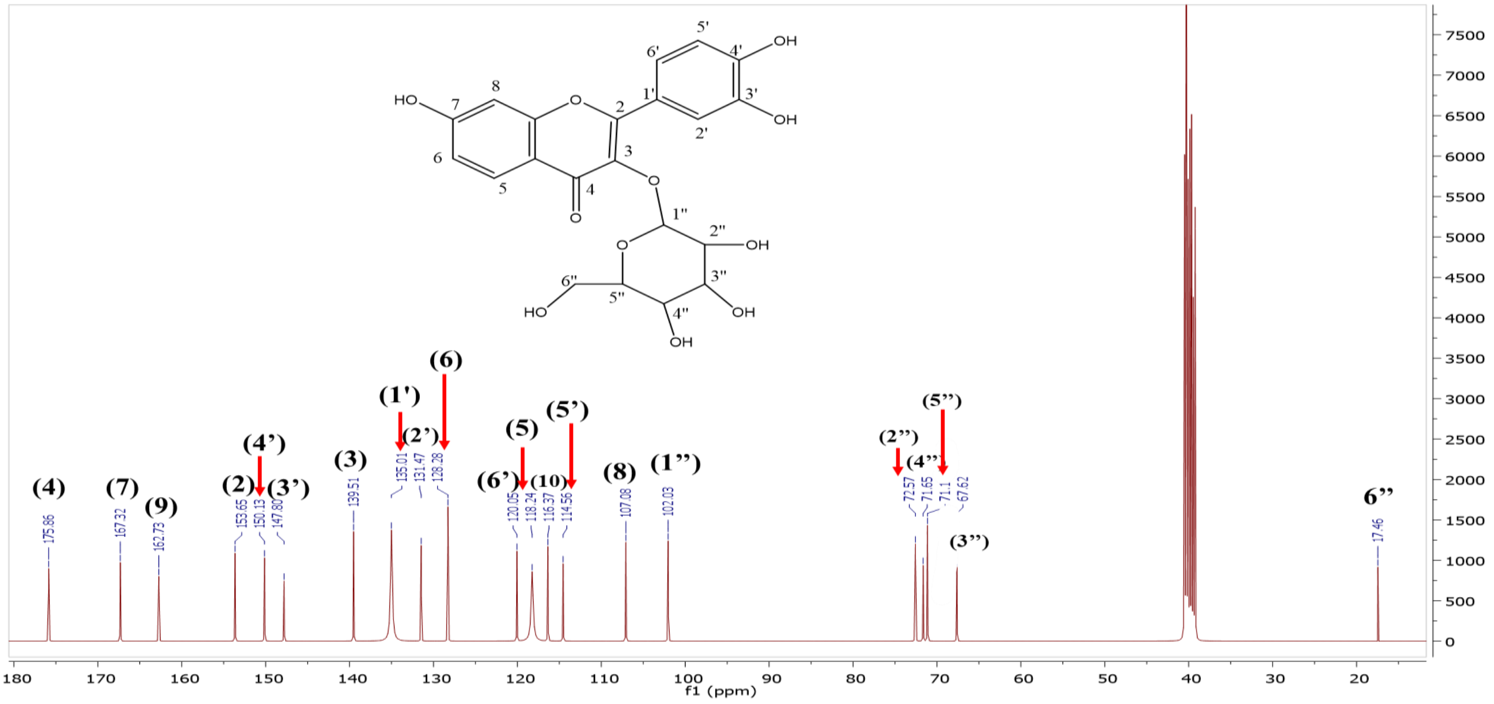

➢ **Fig. S.6:** ^{13}C NMR spectrum of Compound **2** (**Fisetin-3-*O*-rhamnoside).**

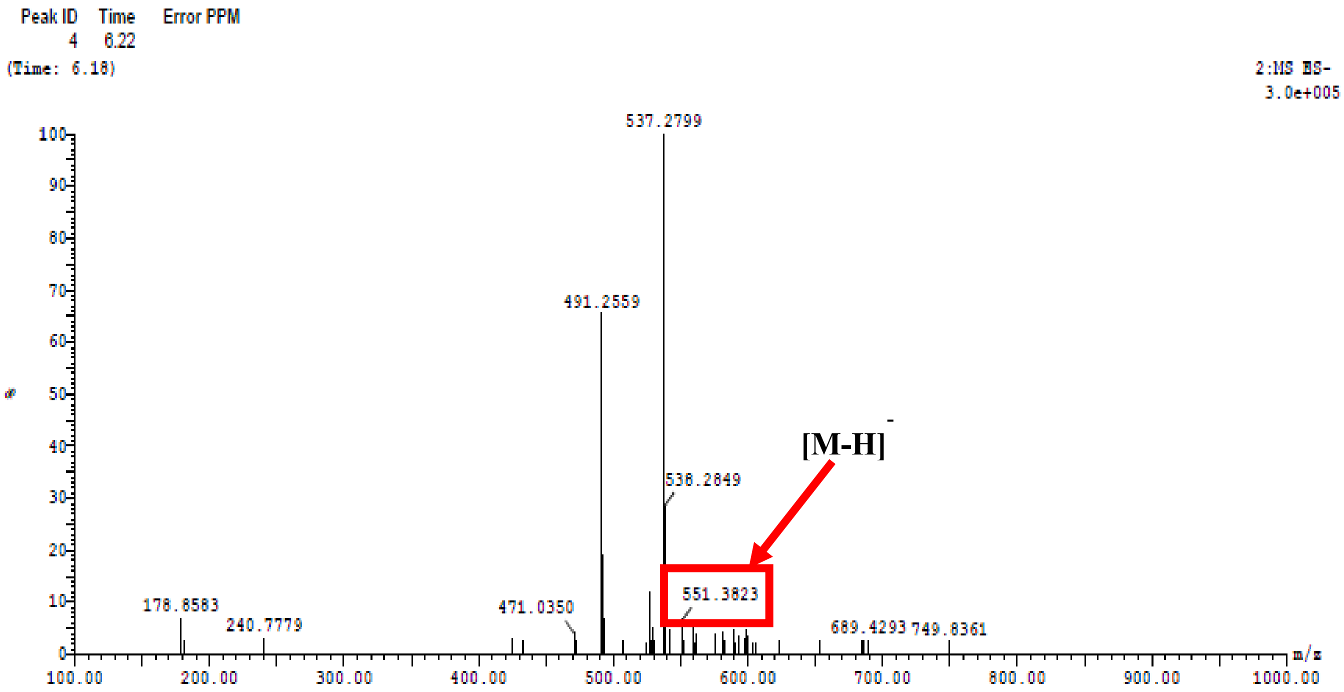

➢ **Fig. S.7**: UPLC-ESI-MS chromatogram of Compound **3** (**Alangilignoside D).**

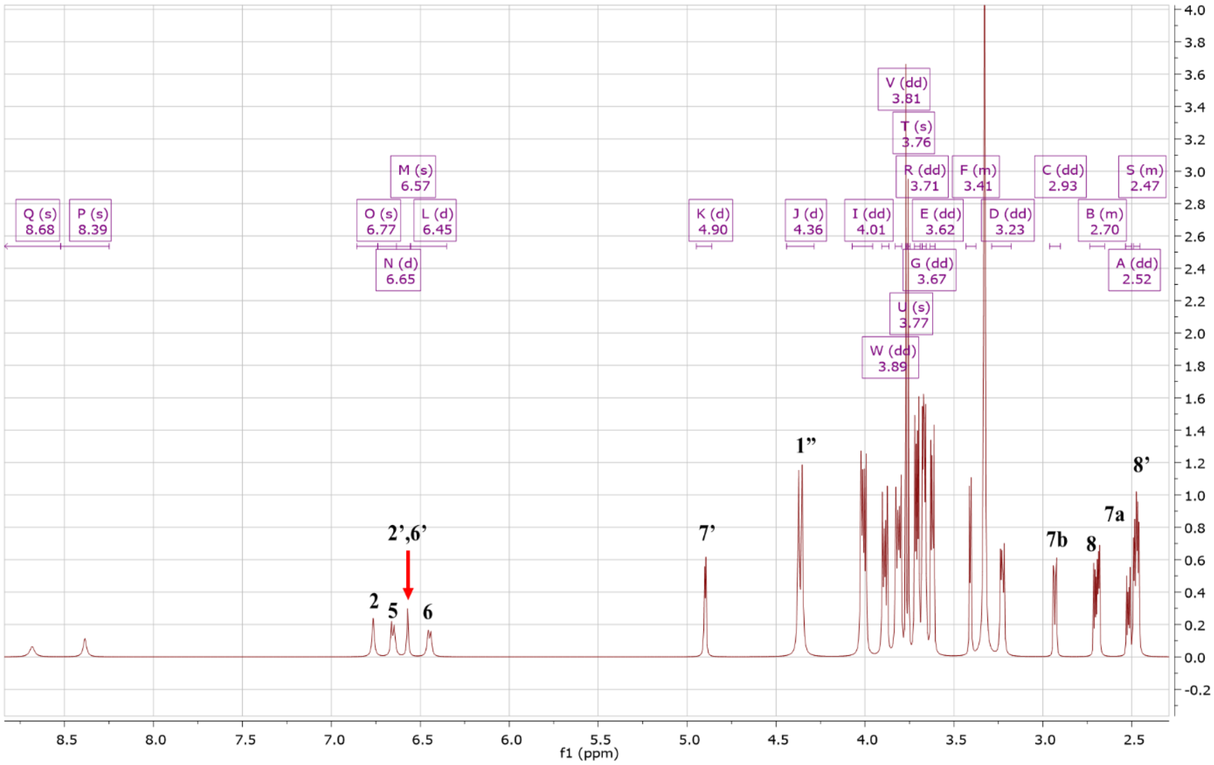

➢ **Fig. S.8:(A)** ^{1}H NMR total Spectrum of Compound **3 (Alangilignoside D).**

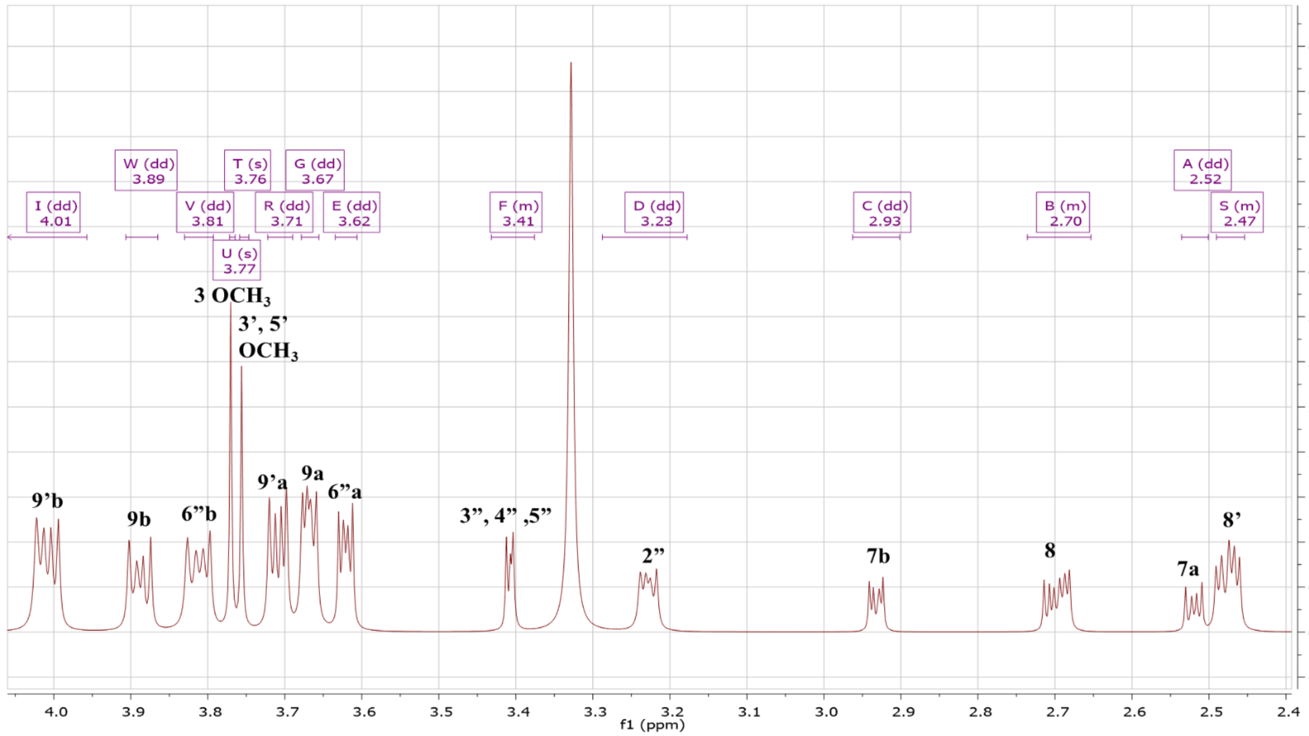

➢ **Fig. S.8: (B)** ^{1}H NMR spectrum of Compound **3** (expansion from 2.4- 4.2 ppm).

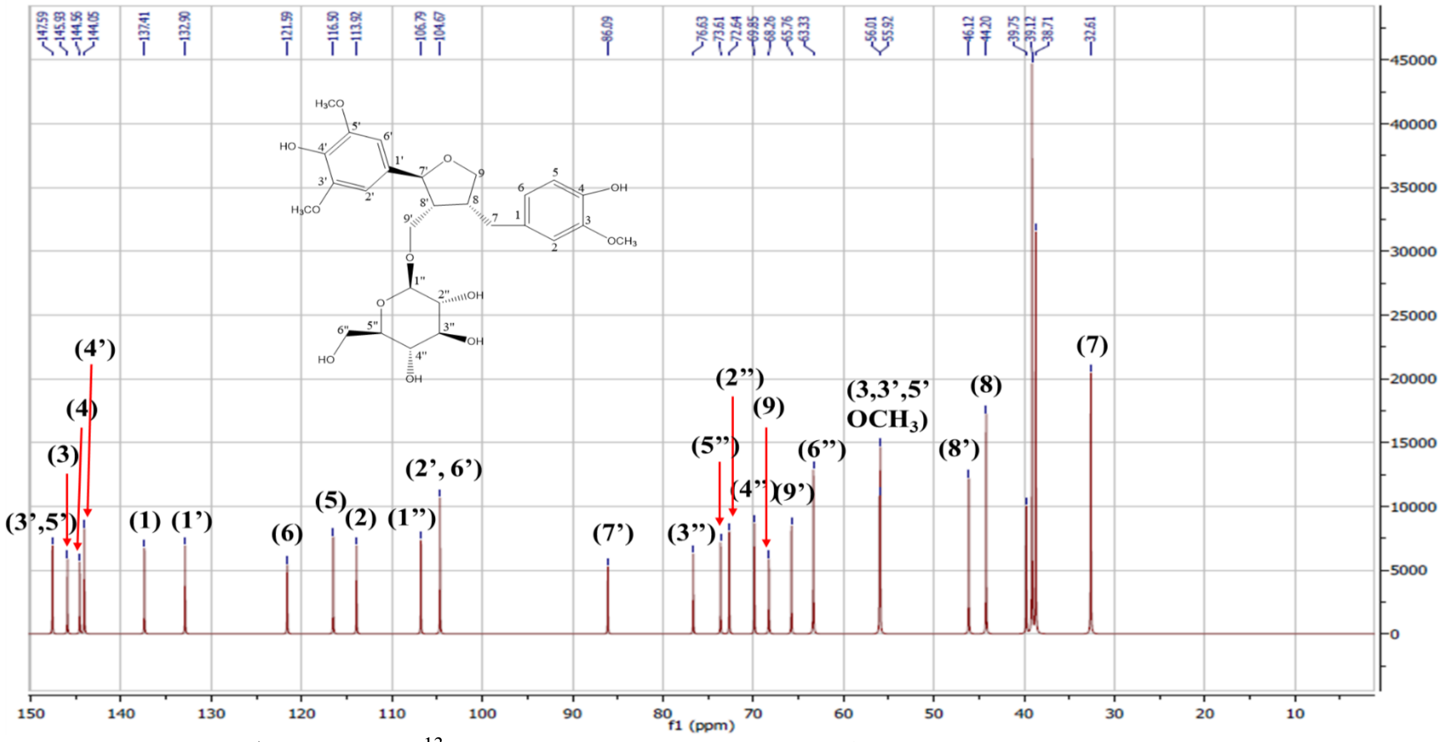

➢ **Fig. S.9**: ^{13}C NMR spectrum of Compound **3** (**Alangilignoside D**).

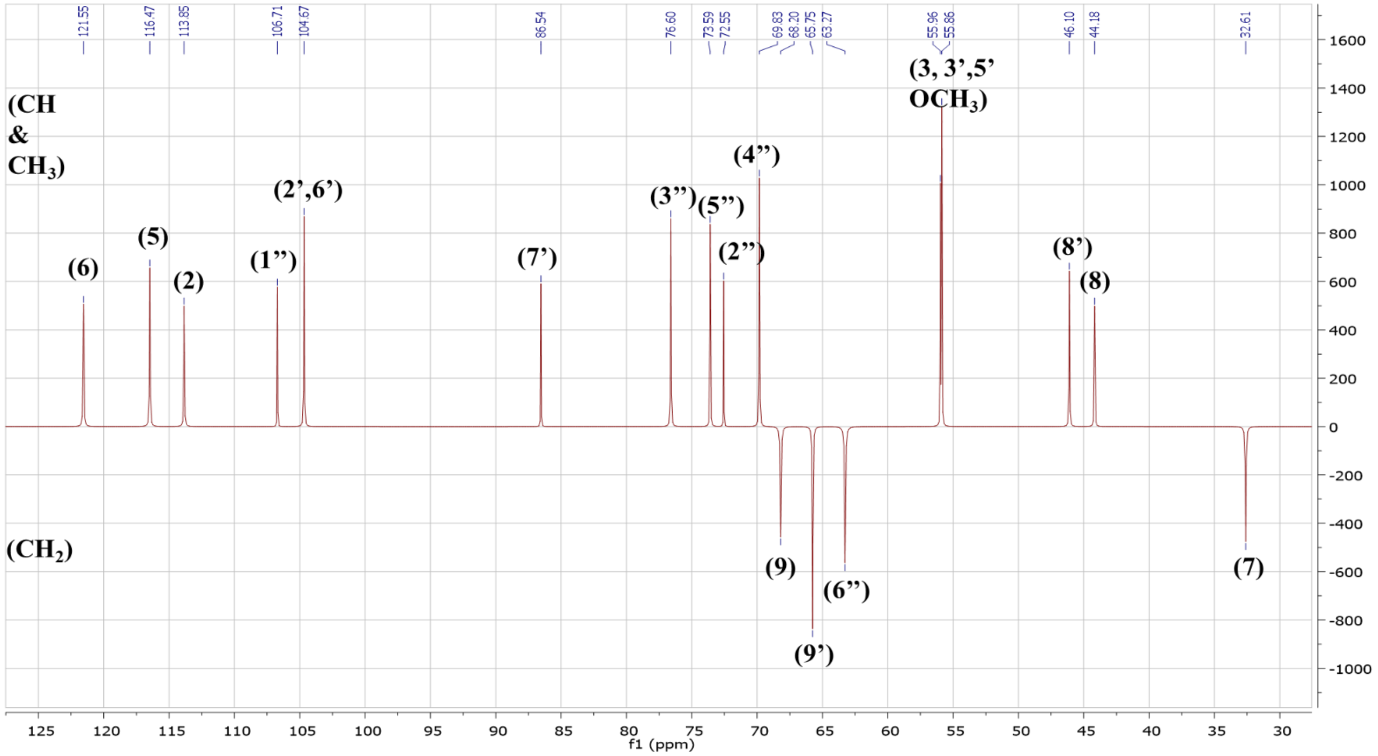

➢ **Fig. S.10**: DEPT-135 NMR spectrum of Compound **3** (**Alangilignoside D**).

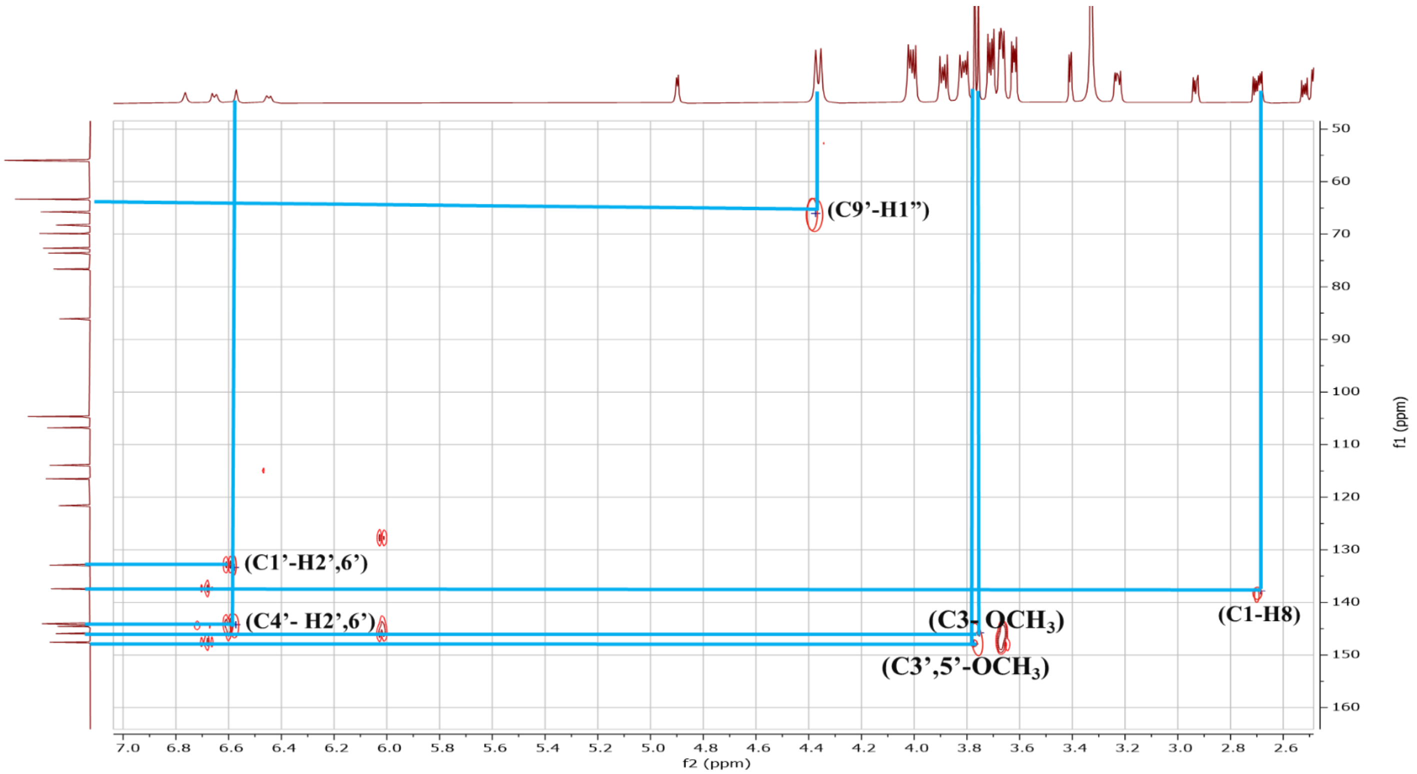

➢ **Fig. S.11**: HMBC NMR total spectrum of Compound **3** (**Alangilignoside D**)

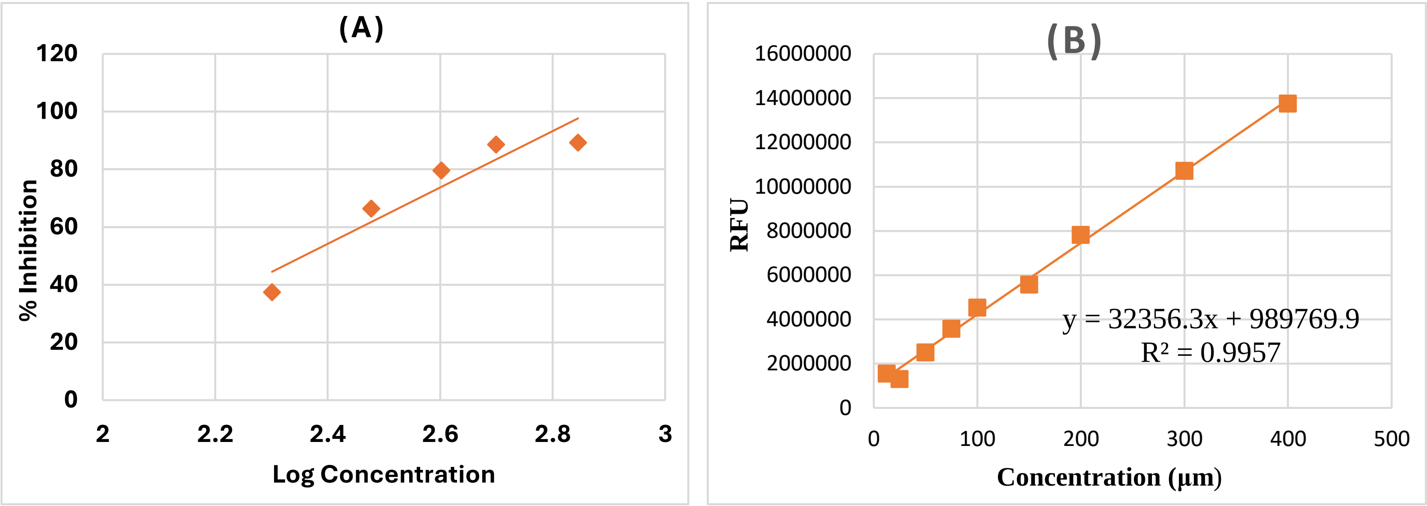

➢ **Figure S.12: (A)** DPPH radical scavenging activity of the total methanolic extract of *P. dulce* leaves. **(B)** ORAC assay of the total methanolic extract of *P. dulce* leaves.

log(inhibitor) vs. normalized response	
Best-fit values	
Log IC_{50}	0.3118
Slope	-0.4807
IC_{50}	2.050
95% CI (profile likelihood)	
Log IC_{50}	0.2010 to 0.4237
Slope	-0.5378 to -0.4308
IC_{50}	1.588 to 2.653
R squared	0.98
IC_{50}: half maximum cytotoxic effect, CI: confidence interval.	

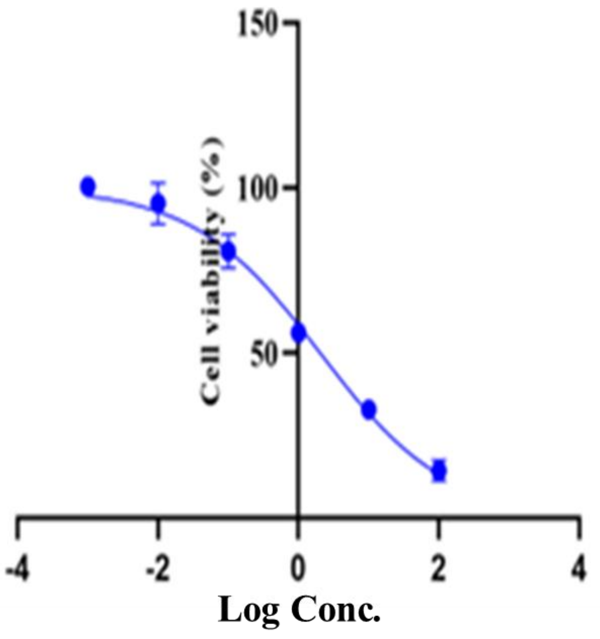

➢ **Figure S.13:** Linear regression curve illustrating the log dose of *P. dulce* extract *and* IC_{50} calculation.

ANOVA summary	
**F**	929.12
***p* value**	<0.0001
***p* value summary**	****
**Significant diff. among means (*p* < 0.05)**	Yes
**R squared**	0.9976

Bar chart showed a high significant difference between the cell viability (%) in HeLa cells treated with 100 µg/mL of the extract and untreated cells ($p < 0.0001$). Data are presented as mean and SD, *: significant difference compared to untreated cells.

- **Figure S.14:** ANOVA test showing the cell viability (%) significant difference between HeLa cells treated with 100 µg/mL of *P. dulce* extract and untreated cells.

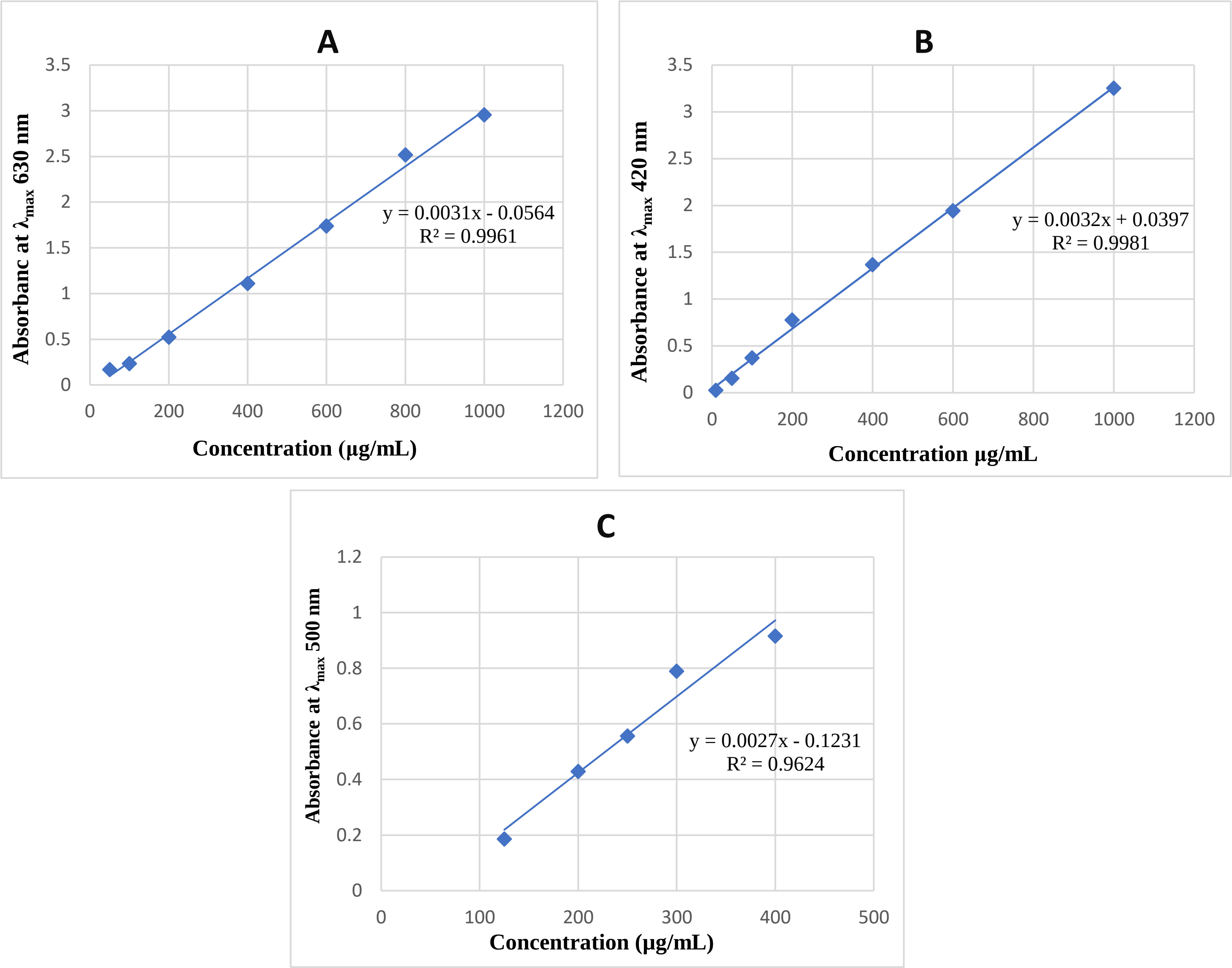

➢ **Figure S.15: A:** Calibration curve of total phenolics. **B:** Calibration curve of total flavonoids. **C:** Calibration curve of total tannins.

➢ **Table S.1:** The wound width and closure percent of the methanolic extract of *P. dulce* leaves vs control.

Time (hrs.)	Average wound width (mm)		Wound closure %		Migration Rate (mm/hr.)	
	P. dulce	**Control**	***P. dulce***	**Control**	***P. dulce***	**Control**
0	3.3 ± 0.08	3.2133 ± 0.006	0	0	0	0
24	2.3 ± 0.386	1.6367 ± 0.578	31.25	49.1	0.004	0.065
48	1.7 ± 0.269	0.3067 ± 0.531	48.48	90.45	0.033	0.061
72	0.7 ± 0.6	0	78.78	100	0.036	0.045

Table S.2: Unpaired t-test comparing the cell viability% between cancer cells treated with the extract IC_{50} of *P. dulce* and the untreated cells, in each cell line.

Unpaired t-test	Mean difference	95% CI	*p* value
A-549 vs. A5-49 + *P. dulce*	-13.60	-17.70 to -9.49	0.0001
WI-38 vs. WI-38 + *P. dulce*	-2.03	-5.06 to 1.002	0.050
Hela vs. Hela + *P. dulce*	86.53	82.36 to 90.69	< 0.0001
Saos-2 vs. Saos-2+ *P. dulce*	-7.70	-10.43 to -4.96	0.0023
MCF-7 vs. MCF-7 + *P. dulce*	39.27	32.95 to 45.58	0.0004
CI: confidence interval			

Table S.3: Tukey's multiple comparisons test comparing the cell viability % between extract-treated HeLa cancer cells with the untreated cells and cisplatin-treated cells.

Tukey's multiple comparisons test	Mean Diff.	95.00% CI	Adjusted *p* value
HeLa vs. *P. dulce* (100µg/mL)	85.83	76.76 to 94.90	< 0.0001 [HS]
HeLa vs. Cisplatin (5µM)	87.92	77.50 to 98.32	< 0.0001 [HS]
P. dulce (100µg/mL) vs. Cisplatin (5µM)	2.08	-1.71 to 5.87	0.5 [NS]
**CI:** confidence interval, **HS:** high significance, **NS:** no significance.			

➢ **Table S.4:** Detailed Phytochemical screening tests:

Chemical Class	Extract	Reagents	Result
Carbohydrates	1 ml	Few drops α-naphthol + few drops H_2SO_4..	Violet ring.
Reducing sugars	1 mL	1 mL water + 5–8 drops of Fehling's (A + B) + boil.	Brick-red precipitate.
Anthraquinones	2 mL	1 ml HCL + 1 ml $FeCl_3$ + chloroform + 1 ml NH_4OH.	Rose red color in the aqueous layer.
Alkaloids	1 mL	Few drops of Dragendorff's reagent.	Reddish orange precipitates or turbidity.
Tannins	1 mL	Ferric chloride ($FeCl_3$ 1%).	Green color.
Terpenoids	1 mL	2 mL chloroform + few drops acetic anhydride + 2 mL H_2SO_4.	Reddish brown ring.
Flavonoids	1 mL	5 mL potassium hydroxide (KOH 50%).	Yellow color.
Sterols	1 mL	2 mL chloroform + few drops acetic anhydride + concentrated H_2SO_4.	Reddish brown ring + upper green color.
Cardiac glycosides	2 mL	1 mL lead acetate + 1 ml $NaHPO_4$ drop $FeCl_3$ (5%) + 1 mL concentrated H_2SO_4.	Orange color.
Saponins	2 mL	Vigorous shaking with distilled water (5 min).	Persistent layer of foam.

- **Docking with PDB Pocket (2W3L): Figures (S.16-S.19):**

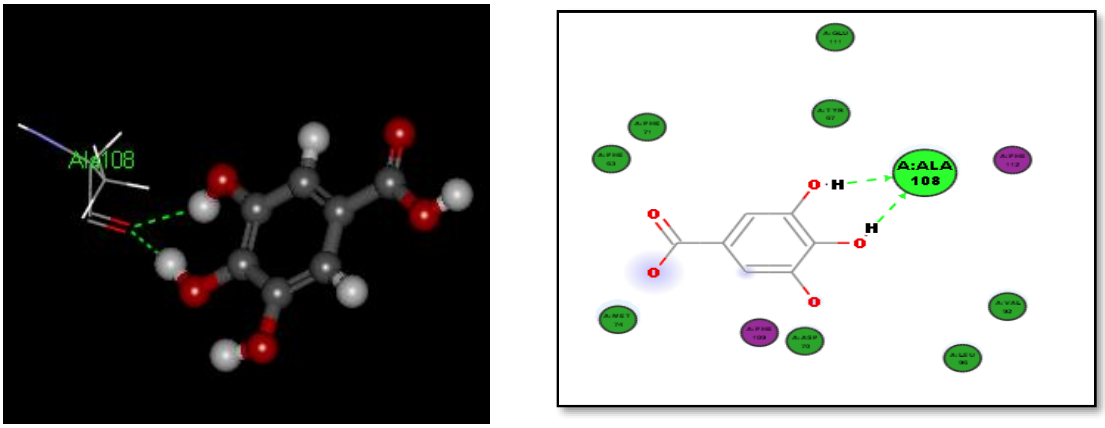

Figure (S.16): Docking of Gallic acid within 2W3L (3D and 2D): 2H-Bonds with ALA108 amino acid.

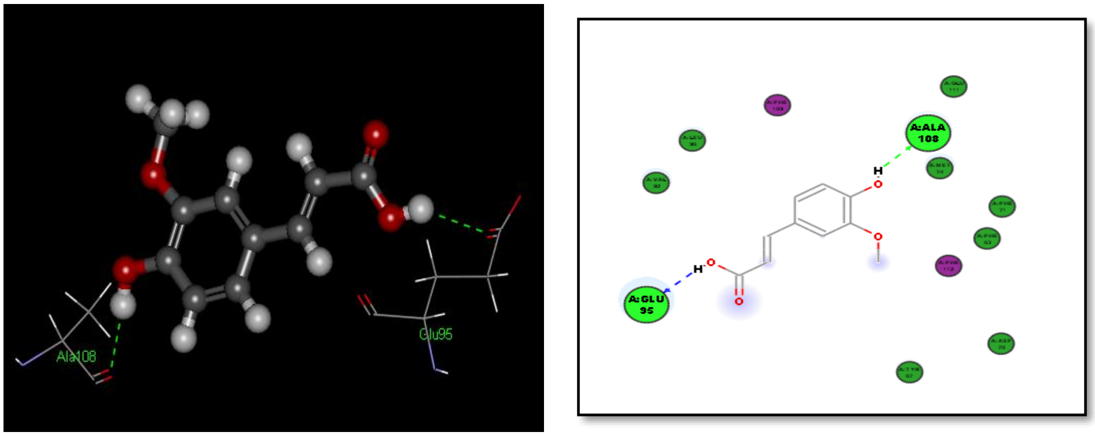

Figure (S.17): Docking of Ferulic acid within 2W3L (3D and 2D): 1H-Bond with GLU95 and 1H-Bond with ALA108 amino acid.

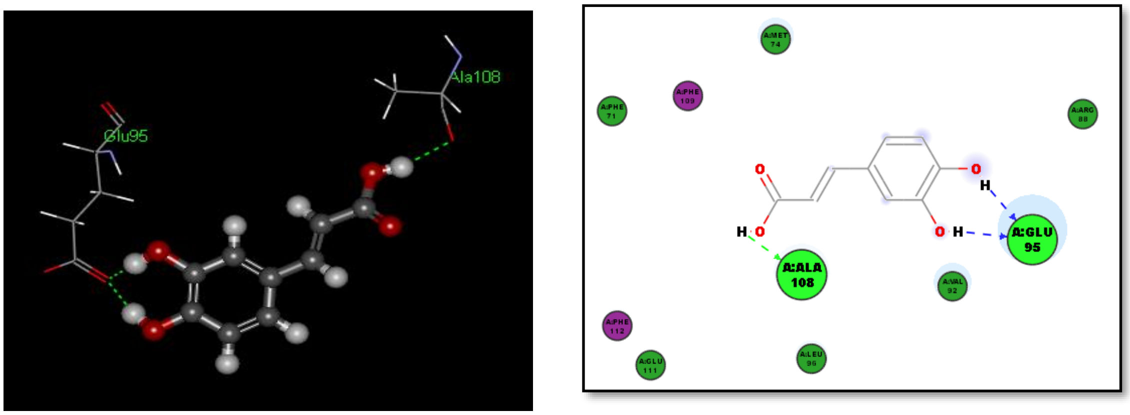

Figure (S.18): Docking of Caffeic acid within 2W3L (3D and 2D): 2H-Bonds with GLU95 and 1H-Bond with ALA108 amino acids.

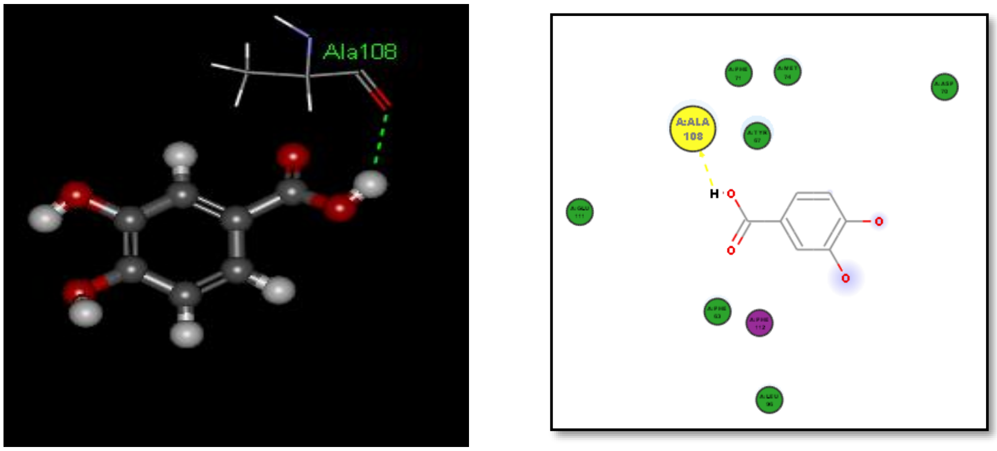

Figure (S.19): Docking of Protocatechuic acid within 2W3L (3D and 2D): 1H-Bond with ALA108 amino acid.

- **Docking with PDB Pocket (1XKK): Figures (S.20-S.23)**

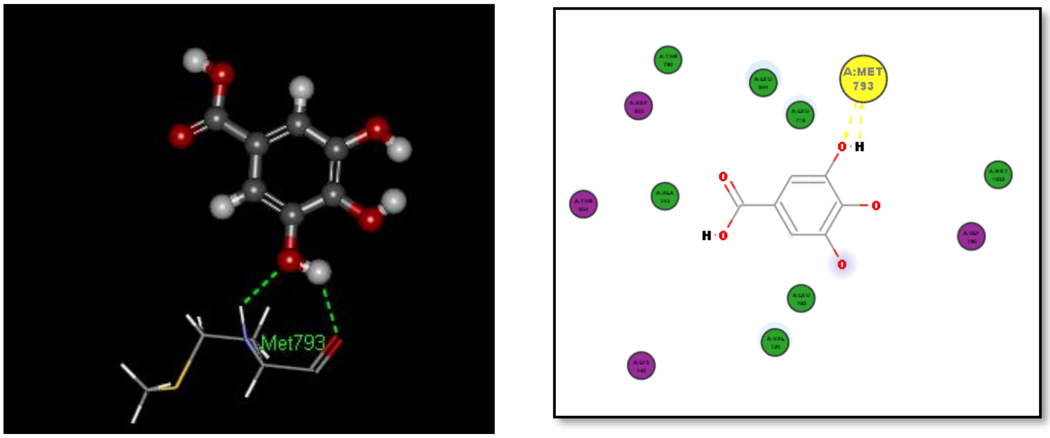

Figure (S.20): Docking of Gallic acid within 1XKK (3D and 2D): 2H-Bonds with MET793 amino acid.

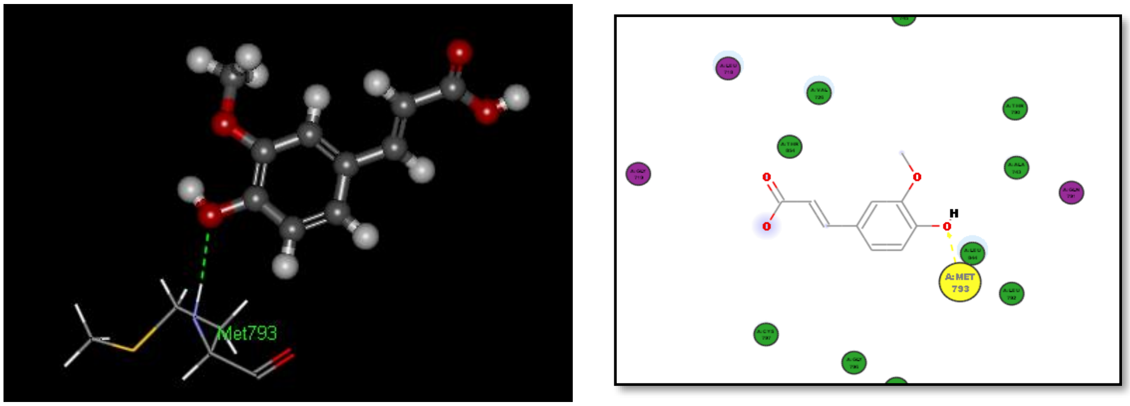

Figure (S.21): Docking of Ferulic acid within 1XKK (3D and 2D): H-Bond with MET793 amino acid.

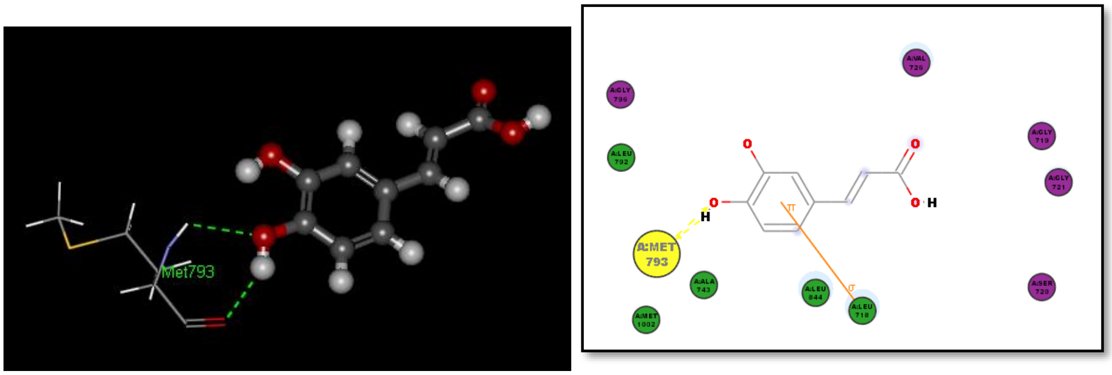

Figure (S.22): Docking of Caffeic acid within 1XKK (3D and 2D): 2H-Bonds with MET793 amino acid.

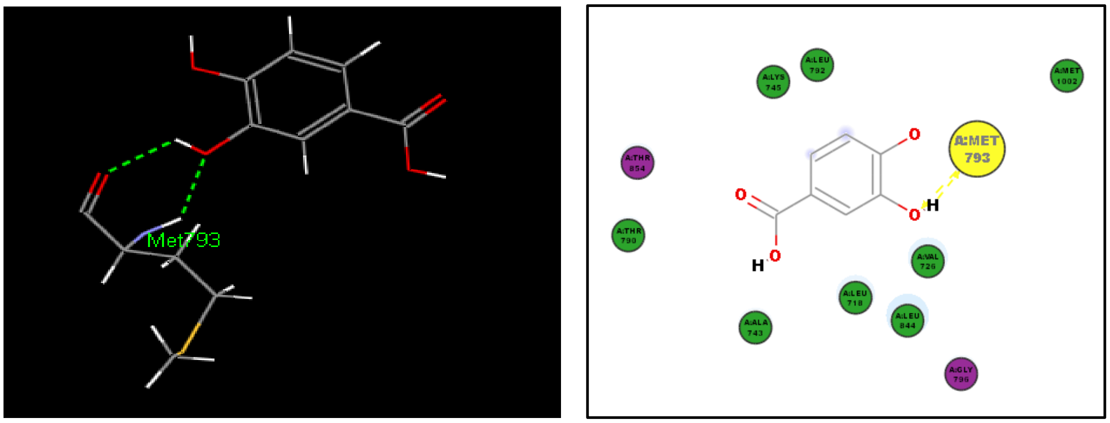

Figure (S.23): Docking of Protocatechuic acid within 1XKK (3D and 2D): 2H-Bonds with MET793 amino acid.
